# COVID-19 mRNA booster vaccine induces transient CD8+ T effector cell responses while conserving the memory pool for subsequent reactivation

**DOI:** 10.1038/s41467-022-32324-x

**Published:** 2022-08-08

**Authors:** Matthias Reinscheid, Hendrik Luxenburger, Vivien Karl, Anne Graeser, Sebastian Giese, Kevin Ciminski, David B. Reeg, Valerie Oberhardt, Natascha Roehlen, Julia Lang-Meli, Kathrin Heim, Nina Gross, Christina Baum, Siegbert Rieg, Claudius Speer, Florian Emmerich, Susanne Breisinger, Daniel Steinmann, Bertram Bengsch, Tobias Boettler, Georg Kochs, Martin Schwemmle, Robert Thimme, Christoph Neumann-Haefelin, Maike Hofmann

**Affiliations:** 1 Department of Medicine II (Gastroenterology, Hepatology, Endocrinology and Infectious Diseases), Freiburg University Medical Center, Faculty of Medicine, University of Freiburg, Freiburg, Germany; 2 Faculty of Biology, University of Freiburg, Freiburg, Germany; 3 IMM-PACT, Faculty of Medicine, University of Freiburg, Freiburg, Germany; 4 Institute of Virology, Freiburg University Medical Center, Faculty of Medicine, University of Freiburg, Freiburg, Germany; 5 Department of Nephrology, University of Heidelberg, Heidelberg, Germany; 6 Molecular Medicine Partnership Unit Heidelberg, European Molecular Biology Laboratory, Heidelberg, Germany; 7 Institute for Transfusion Medicine and Gene Therapy, Freiburg University Medical Center, Faculty of Medicine, University of Freiburg, Freiburg, Germany; 8 Occupational Medical Service, Freiburg University Medical Center, Faculty of Medicine, University of Freiburg, Freiburg, Germany; 9 Signalling Research Centres BIOSS and CIBSS, University of Freiburg, Freiburg, Germany; 10 Berta-Ottenstein Programme, Faculty of Medicine, University of Freiburg, Freiburg, Germany

**Keywords:** RNA vaccines, Immunological memory, Cytotoxic T cells, SARS-CoV-2

## Abstract

Immunization with two mRNA vaccine doses elicits robust spike-specific CD8^+^ T cell responses, but reports of waning immunity after COVID-19 vaccination prompt the introduction of booster vaccination campaigns. However, the effect of mRNA booster vaccination on the spike-specific CD8^+^ T cell response remains unclear. Here we show that spike-specific CD8^+^ T cells are activated and expanded in all analyzed individuals receiving the 3^rd^ and 4^th^ mRNA vaccine shots. This CD8^+^ T cell boost response is followed by a contraction phase and lasts only for about 30-60 days. The spike-specific CD8^+^ T memory stem cell pool is not affected by the 3^rd^ vaccination. Both 4^th^ vaccination and breakthrough infections with Delta and Omicron rapidly reactivate CD8^+^ T memory cells. In contrast, neutralizing antibody responses display little boost effect towards Omicron. Thus, COVID-19 mRNA booster vaccination elicits a transient T effector cell response while long-term spike-specific CD8^+^ T cell immunity is conserved to mount robust memory recall targeting emerging variants of concern.

## Abstract (web summary)

Vaccines induce beneficial immunity for COVID-19, but immune waning prompts boosting vaccination. Here, the authors show that a third, boosting dose of COVID-19 mRNA vaccine induces transient CD8 + T effector cell response while conserving the CD8 memory T cell pool, thereby permitting reactivation of spike-specific CD8 + T cells upon breakthrough infection or 4th vaccination.

## Introduction

More than two years after the outbreak of the SARS-CoV-2 pandemic, constantly emerging variants of concern (VOCs) still fuel the pandemic situation and vaccination is considered to be an important measure to accelerate the transition from the pandemic into the endemic stage^1^. mRNA vaccines against SARS-CoV-2 have been proven to rapidly induce protection against symptomatic COVID-19 and death e.g., by inducing robust antibody and T cell responses^2–5^. However, reports of waning humoral immunity^6–8^ and high SARS-CoV-2 infection incidences with the VOC Omicron (B.1.1.529) prompted to large booster vaccination campaigns. Immediate beneficial effects of these booster immunizations were detectable with respect to the neutralization capacity of the humoral response^9^.

The effect of mRNA booster vaccination on the spike-specific CD8^+^ T cell response remains, however, unclear. In particular, there is little information about the kinetics and effects on long-term immunity and recall responses in breakthrough infections.

Here, we show that after the 3^rd^ mRNA vaccine dose there is a substantial but transient booster effect on spike-specific CD8^+^ T cell frequencies without marked effect on the memory response. In contrast, only a minor boost effect on the neutralizing antibody response against Omicron is detectable after the 3^rd^ vaccine dose. Hence, mRNA vaccines elicit robust and durable spike-specific CD8^+^ T cell responses already after basic immunization with minor effects of the booster immunization on long-term CD8^+^ T cell immunity.

## Results

### Transient spike-specific CD8^+^ T cell booster responses

**Fig. 1:**
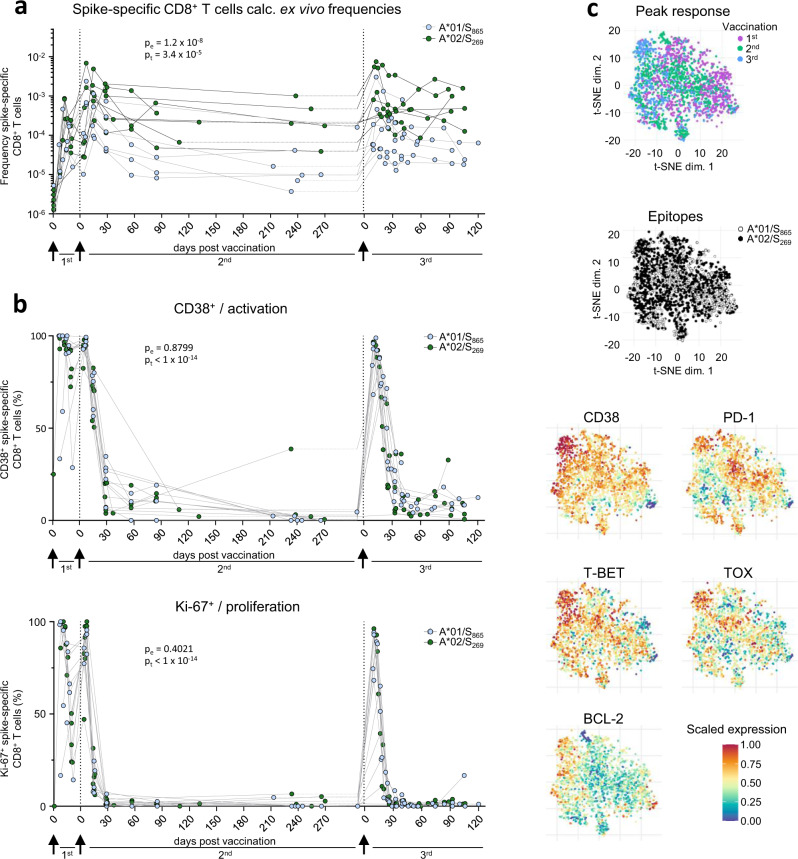
Cellular and humoral effector response after the 3^rd^ vaccine dose. Calculated ex vivo frequencies of spike-specific CD8^+^ T cells throughout 1^st^, 2^nd^ and 3^rd^ vaccination (**a**). Ki-67,and CD38 expression within spike-specific non-naïve CD8^+^ T cells (**b**). t-SNE representation of flow cytometry data comparing A*01/S_865_- and A*02/S_269_-specific CD8^+^ T cells at peak response after 1^st^, 2^nd^ and 3^rd^ vaccination. Expression levels of CD38, T-BET, BCL-2, PD-1 and TOX are depicted (**c**). Statistical significance was determined by two-way ANOVA with main model (a, b) comparing the effects of targeted epitopes (p_e_) and of the time course (p_t_). Source data are provided as a Source Data file.

**Fig. 2:**
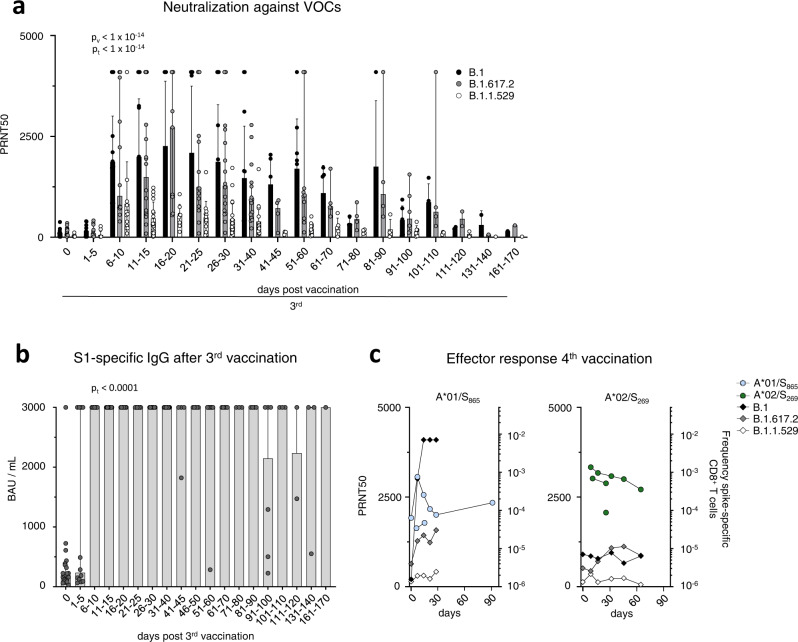
Humoral response after the 3^rd^ vaccine dose. Antibody neutralization activity as 50% plaque reduction neutralization tests (PRNT50) for SARS-CoV-2 variants B.1, B.1.617.2 and B.1.1.529 after 3^rd^ vaccination. Median values are depicted with 95% confidence interval error bars with *n* = 28 individual tested (**a**). Quantification of serum anti-SARS-CoV-2 spike IgG levels following 3^rd^ vaccination with *n* = 26 individuals tested (**b**). Calculated ex vivo frequencies of A*01/S_865_- (left) and A*02/S_269_-specific (right) CD8^+^ T cells, with antibody neutralization activity after 4^th^ vaccination (**c**). Median values are depicted with 95% confidence interval error bars. Statistical significance was calculated by Kruskal-Wallis test (a) comparing the effects of time course (p_t_ < 1×10^−14^). Source data are provided as a Source Data file.

To assess the longevity of boosted adaptive effector responses, we longitudinally collected sera and peripheral blood mononuclear cells (PBMCs) from 13 volunteers (Supplementary Table 1) before and after 3^rd^ mRNA vaccination with Comirnaty/Pfizer-Biontech or Spikevax/Moderna (Supplementary Fig. 1a). All volunteers included in this cohort did not have a history of SARS-CoV-2 infection and received their vaccinations with a three-week interval between 1^st^ and 2^nd^ and six-to-nine-months interval between 2^nd^ and 3^rd^ dose. First, we analyzed the kinetics of the spike-specific CD8^+^ T cell response targeting the A*01/S_865_ (*n* = 7) or A*02/S_269_ epitope (*n* = 8). A*01/S_865_- and A*02/S_269_-specific CD8^+^ T cells are part of a broader CD8^+^ T cell response targeting the spike protein after mRNA vaccination and dominant with respect to the restricting HLA class I allele^3,10^. The targeted epitopes are highly conserved in all VOCs, including the Omicron variants BA.1 and BA.2 (Supplementary Fig. 1b) but not in common cold coronaviruses^3^. To increase the detection sensitivity of spike-specific CD8^+^ T cells targeting single epitopes, we performed peptide-loaded MHC-class I tetramer-based enrichment^3,11^ (Supplementary Fig. 1c). Spike-specific CD8^+^ T cell frequencies had already reached their set point approx. 60 days after the 2^nd^ dose and were rapidly boosted with peak expansion one week after the 3^rd^ dose in all tested donors (Fig. 1a). Robust boosting of the spike-specific CD8^+^ T cell response by the 3^rd^ dose was also reflected by high CD38, Ki-67 and T-BET expression and thus by rapid activation, strong proliferation and substantial induction of an effector cell program (Fig. 1b and Supplementary Fig. 2a). However, within 30-60 days the circulating spike-specific CD8^+^ T cell frequency, activation, proliferation and effector cell differentiation decreased rapidly to a similar level as before the 3^rd^ dose (Fig. 1a, b and Supplementary Fig. 2a), thus following the same kinetics as after the 2^nd^ dose. Similarities between the spike-specific CD8^+^ T cell response after the 2^nd^ and 3^rd^ dose, respectively, are also supported by t-distributed stochastic neighborhood embedding (t-SNE) and pseudotemporal diffusion analysis of T cell phenotypes including CD38, T-BET, BCL-2, PD-1, and TOX expression (Fig. 1c and Supplementary Fig. 2b, c). Phenotypic differences (e.g., in T-BET and TOX expression) were detectable between the spike-specific CD8^+^ T cell response after the 1^st^ dose compared to the 2^nd^ and 3^rd^ doses and only minor differences were apparent with respect to the targeted epitopes A*01/S_865_ or A*02/S_269_ (Fig. 1c and Supplementary Fig. 2b, c). With respect to the neutralizing antibody response, the boost effect clearly depended on the VOC (Fig. 2a and Supplementary Fig. 2b; *p* < 0.0001). In particular, neutralization was only slightly increased against the Omicron variant BA.1 (B.1.1.529) by the 3^rd^ dose, while neutralization of the parental B.1 strain and Delta (B.1.617.2) peaked after approx. 3 weeks followed by a decrease to baseline neutralization after 4 months (Fig. 2a). Of note, after the 4^th^ dose similar kinetics (Fig. 2c) and dynamics (Supplementary Fig. 3a–c) of spike-specific CD8^+^ T cell and neutralizing antibody responses were detectable. Hence, after booster vaccination, there is a transient mRNA vaccine-associated spike-specific CD8^+^ T effector cell response and neutralizing antibody boost lasting about 2 and 4 months, respectively.

### Stable T memory cell response irrespective of booster immunization

**Fig. 3:**
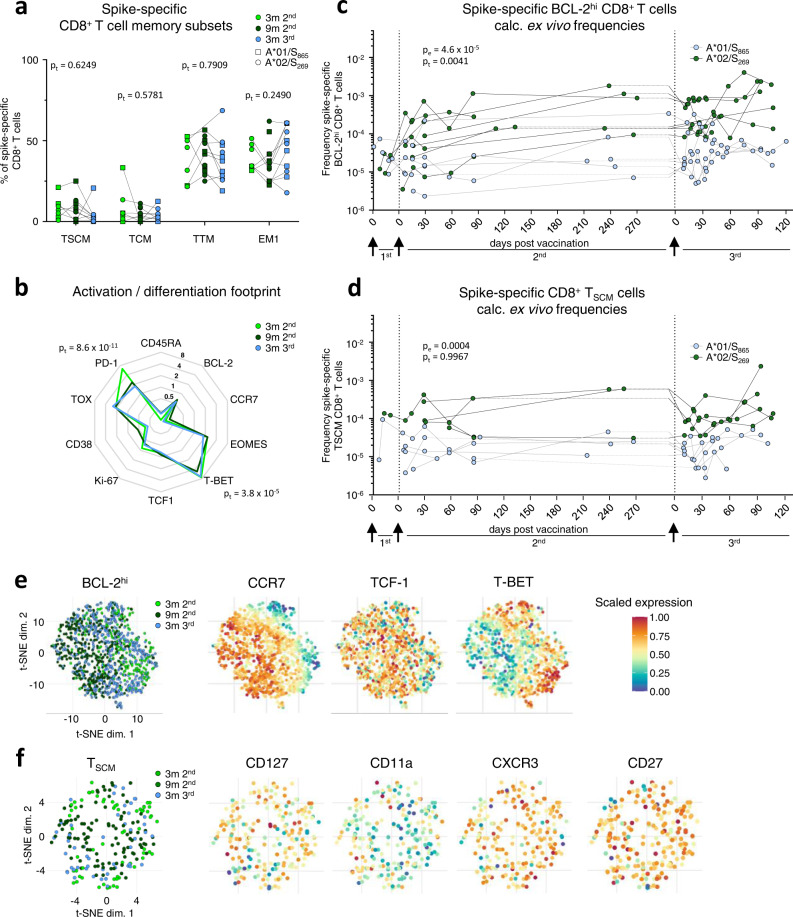
Spike-specific CD8^+^ T memory cells before and after the 3^rd^ vaccine dose. Composition of spike-specific CD8^+^ T cell memory subsets at 3 (*n* = 7) and 9 (*n* = 11) months after 2^nd^, and 3 (*n* = 11) months after 3^rd^ vaccination (**a**). Molecule expression of spike-specific non-naïve CD8^+^ T cells at 3 (*n* = 8) and 9 (*n* = 12) months after 2^nd^, and 3 (*n* = 11) months after 3^rd^ vaccination normalized to bulk naïve CD8^+^ T cells (**b**). Calculated ex vivo frequencies of BCL-2^hi^ (**c**) and T_SCM_ (**d**) spike-specific CD8^+^ T cells. t-SNE representation of BCL-2^hi^ (**e**) and T_SCM_ (**f**) spike-specific CD8^+^ T cells at 3 and 9 months after 2^nd^, and 3 months after 3^rd^ vaccination. Expression levels of CCR7, TCF-1 and T-BET are depicted for BCL-2^hi^, and CD127, CD11a, CXCR3 and CD27 are depicted for T_SCM_ spike-specific CD8^+^ T cells. Statistical significance was calculated by two-way ANOVA with full model and Tukey’s test for multiple comparison (**a**, **b**) to examine the effect of sampling time points (p_t_) on memory subsets and marker expression, and two-way ANOVA with main model (**c**, **d**) to compare the effects of targeted epitopes (p_e_) and time course (p_t_). Source data are provided as a Source Data file.

To investigate the effect of booster immunization on long-term spike-specific CD8^+^ T cell immunity, we comparatively analyzed the spike-specific CD8^+^ T cell response after three and nine months post 2^nd^ dose versus three months after the 3^rd^ dose. The memory T cell subset distribution (Fig. 3a and Supplementary Fig. 4a, b) and activation/differentiation footprint (Fig. 3b and Supplementary Fig. 4c) of spike-specific CD8^+^ T cells were similar at the tested time points, indicating stable memory features without a clear effect of the 3^rd^ dose. Of note, during the effector phase after the 3^rd^ vaccine shot, a transient subset redistribution was detectable (Supplementary Fig. 5a–c). To more precisely quantify and characterize the T cell memory compartment, we analyzed on the one hand, spike-specific CD8^+^ T cells highly expressing BCL-2 (BCL-2^hi^) to map the overall memory pool irrespective of certain subsets and on the other hand, focused on T memory stem cells (T_SCM_, defined by expression of CD45RA, CCR7, CD27, CD28 and CD95; Supplementary Fig. 4a) that are essential for long-term T cell immunity^12^. Spike-specific BCL2^hi^ CD8^+^ T cells and CD8^+^ T_SCM_ cells were induced after the 1^st^ dose and their frequencies remained stable after the 2^nd^ dose throughout the subsequent vaccination (Fig. 3c, d). t-SNE analysis revealed that spike-specific BCL-2^hi^ CD8^+^ T cells showed minor phenotypic differences such as higher T-BET expression 3 months post 2^nd^ and 3^rd^ dose compared to 9 months after the 2^nd^ dose (Fig. 3e and Supplementary Fig. 5d) probably reflecting a more resting state at later time points post immunization. In contrast, phenotypic characteristics of CD8^+^ T_SCM_ remained completely stable, indicated by a complete intermingling within the t-SNE analysis (Fig. 3f). Furthermore, the spike-specific CD8^+^ T cell memory pool was also conserved during and after the 4^th^ dose (Supplementary Fig. 6a–c). Thus, these results indicate that booster immunizations give rise to a rapid effector T cell response that is based on a stable T cell memory pool as illustrated by a subset of spike-specific CD8^+^ T cells expressing high levels of BCL-2 in parallel to T-BET^hi^, PD-1^hi^, TOX^+^ subsets at peak expansion after booster immunization (Supplementary Fig. 3a).

### Conserved spike-specific CD8^+^ T cell recall capacity

**Fig. 4:**
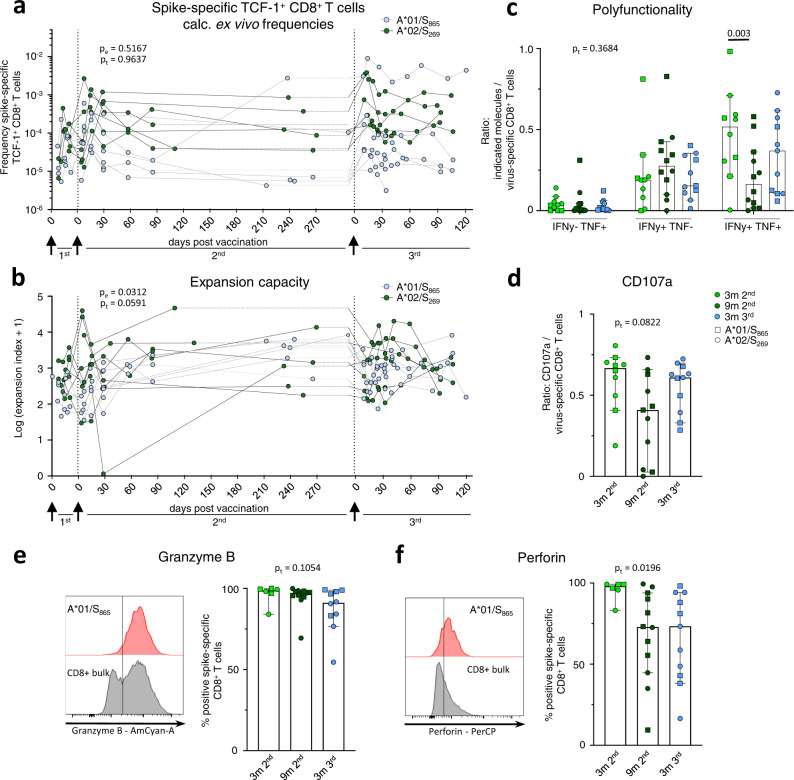
Reactivation capacity of spike-specific CD8^+^ T cells before and after the 3^rd^ vaccine dose. Calculated ex vivo frequencies of TCF-1^+^ non-naive spike-specific CD8^+^ T cells (**a**). Expansion capacity of spike-specific CD8^+^ T cells over 14 days of in vitro expansion throughout 1^st^, 2^nd^ and 3^rd^ vaccination (**b**). Percentage of IFNγ and TNF (**c**), and CD107a (**d**) producing CD8^+^ T cells upon peptide stimulation related to the percentage of spike-specific CD8^+^ T cells from all CD8^+^ T cells at 3 (*n* = 10) and 9 (*n* = 12 for c, *n* = 11 for d) months post 2^nd^, and 3 (*n* = 11) months post 3^rd^ vaccination after in vitro expansion. Median values are depicted with 95% confidence interval error bars. Expression of Granzyme B (**e**) and Perforin (**f**) of spike-specific CD8^+^ T cells at 3 (*n* = 6) and 9 (*n* = 12) months post 2^nd^, and 3 months (*n* = 10 for **e**, *n* = 11 for **f**) post 3^rd^ vaccination after in vitro expansion with representative histograms. Median values are depicted with 95% confidence interval error bars. Statistical significance was calculated by two-way ANOVA with main model (**a**, **b**) to compare the effects of targeted epitopes (p_e_) and time course (p_t_), two-way ANOVA with full model and Tukey’s multiple comparison test (**c**), and Kruskal-Wallis test (**d**, **e**, **f**) to examine the effect of sampling time on functionality (p_t_). Source data are provided as a Source Data file.

Next, we analyzed the recall capacity of spike-specific CD8^+^ T cells after the 3^rd^ dose. For this, we stained for TCF-1 expression, a transcription factor indicative of the proliferative capacity of T cells^13^. The frequencies of TCF-1^+^ spike-specific CD8^+^ T cells were stable suggesting a conserved pool of proliferative competent T cells throughout the 2^nd^ and 3^rd^ dose (Fig. 4a). Accordingly, the peptide-specific in vitro expansion capacity of spike-specific CD8^+^ T cells was also conserved before and after the 3^rd^ dose (Fig. 4b and Supplementary Fig. 7a, b). Furthermore, after two weeks of expansion, spike-specific CD8^+^ T cells produced similar amounts of IFNγ and TNF, including co-production irrespective of whether the input cells were obtained before or after the 3^rd^ dose (Fig. 4c and Supplementary Fig.7c, d). Spike-specific production of IFNγ and TNF were assessed in relation to the frequency of spike-specific CD8^+^ T cells after expansion as a measure of the effector function per cell. In order to estimate the cytotoxic potential of spike-specific CD8^+^ T cells after in vitro expansion, we analyzed degranulation as indicated by CD107a expression in relation to the frequency of spike-specific CD8^+^ T cells (Fig. 4d and Supplementary Fig. 7e) and Granzyme B (Fig. 4e) and Perforin (Fig. 4f) expression of spike-specific CD8^+^ T cells. Similar to cytokine production, the cytotoxic potential was also largely conserved with almost all spike-specific CD8^+^ T cells expressing Granzyme B and Perforin. Thus, recall capacity tested in vitro comprising expansion capacity, differentiation to cytokine-producing and cytotoxic effector cells is robust and stable before and after 3^rd^ vaccination.

### Recall response in breakthrough infections

**Fig. 5:**
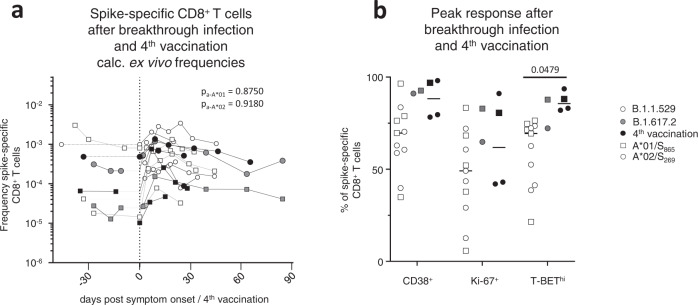
CD8^+^ T effector cell response after breakthrough infection and the 4^th^ vaccine dose. Calculated ex vivo frequencies of spike-specific CD8^+^ T cells before and after breakthrough infection and 4^th^ vaccination (**a**). Proportion of CD38^+^, Ki-67^+^ and T-BET^hi^ within non-naive spike-specific CD8^+^ T cells at peak expansion after breakthrough infection and 4^th^ vaccination with *n* = 11 for Omicron and *n* = 2 for Delta breakthrough infections, and *n* = 4 individuals receiving a 4^th^ vaccination (**b**). Statistical significance was calculated by two-way ANOVA with main model to compare the effects of antigen triggers (p_a_) on epitope-specific T cell frequencies (**a**) and two-way ANOVA with full model and Tukey’s test for multiple comparison (**b**) to examine the effects of antigen triggers on activation marker expression. Source data are provided as a Source Data file.

**Fig. 6:**
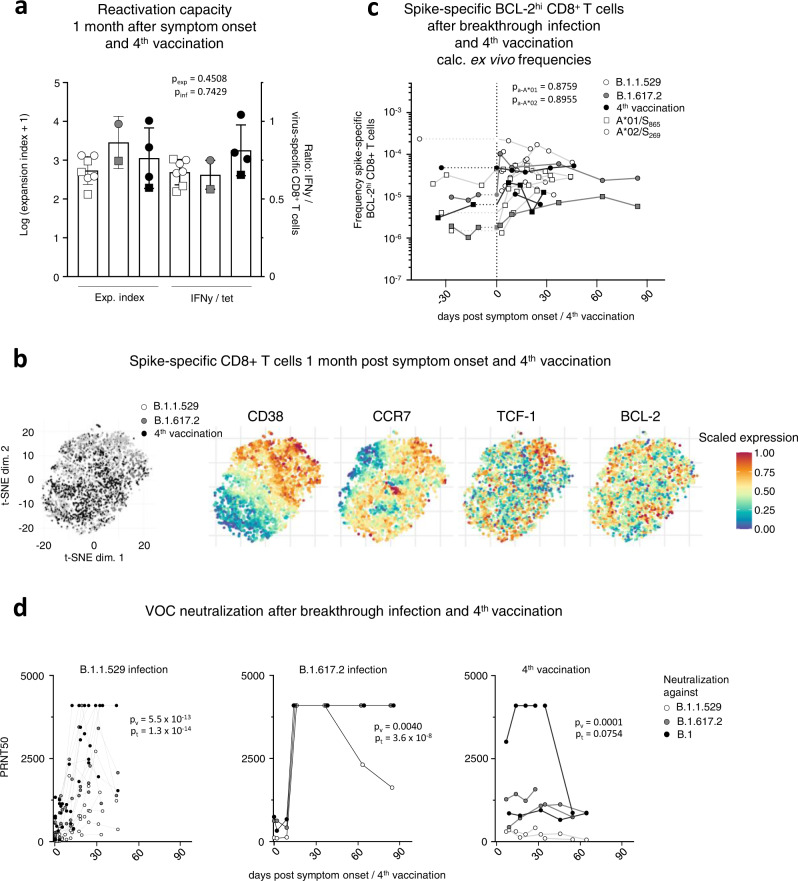
Characterization of CD8^+^ T memory cells after breakthrough infection and the 4^th^ vaccine dose. Reactivation capacity of spike-specific CD8^+^ T cells 1 month after symptom onset and 4^th^ vaccination. Median values are depicted with 95% confidence interval error bars with *n* = 6 for Omicron and *n* = 2 for Delta breakthrough infections, and *n* = 4 individuals receiving a 4^th^ vaccination (**a**). t-SNE representation of spike-specific CD8^+^ T cells 1 month after symptom onset and 4^th^ vaccination. Expression levels of CD38, CCR7, TCF- and BCL-2 are depicted (**b**). Calculated ex vivo frequencies of BCL-2^hi^ non-naïve spike-specific CD8^+^ T cells before and after breakthrough infection and 4^th^ vaccination (**c**). Antibody neutralization activity as 50% plaque reduction neutralization tests (PRNT50) for SARS-CoV-2 variants B.1, B.1.617.2 and B.1.1.529 after breakthrough infection and 4^th^ vaccination with *n* = 11 for Omicron and *n* = 1 for Delta breakthrough infection, and *n* = 2 individuals receiving a 4^th^ vaccination (**d**). Statistical significance was calculated by Kruskal-Wallis test (**a**) to compare the effect of the antigen triggers on expansion (p_exp_) and interferon production (p_inf_) and two-way ANOVA with main model (**c**, **d**) to compare the effects of antigen triggers (p_a_) on epitope-specific T cell frequencies (**c**) or the effects of VOCs (p_v_) and time course (p_t_). Source data are provided as a Source Data file.

To determine the recall capacity in vivo and to compare booster responses of vaccine-elicited CD8^+^ T cell responses after vaccination versus infection, we next analyzed spike-specific CD8^+^ T cells and the neutralizing antibody capacity in breakthrough infections. Specifically, we compared the adaptive effector immune response after the 4^th^ antigen contact, either by the 4^th^ vaccine dose (*n* = 5) or by breakthrough infection with Omicron (*n* = 12) or Delta (*n* = 2) after three mRNA vaccine doses. The spike-specific CD8^+^ T cell response was rapidly and robustly induced and similar after the 4^th^ vaccine dose versus an Omicron or Delta breakthrough infection (Fig. 5a and Supplementary Fig. 8a). Spike-specific CD8^+^ T cells peaked in a classical effector cell response with pronounced expression of CD38, Ki-67 and T-BET (Fig. 5b and Supplementary Fig. 8a–c). Variations within the proportion of spike-specific CD8^+^ T cells expressing these markers may be due to differences in infection time point or time point of symptom onset, which is especially relevant in breakthrough infections with Omicron that are often characterized by mild symptoms in vaccinated individuals. However, within one to two months after breakthrough infection and 4^th^ vaccine dose, a fully functional early T cell memory was present with similar reactivation capacities comprising expansion, cytokine production and degranulation (Fig. 6a and Supplementary Fig. 9a). Furthermore, phenotypic characteristics of early spike-specific CD8^+^ T memory cells were similar after breakthrough infections and 4^th^ vaccine dose as depicted by t-SNE analysis including CD38, CCR7, TCF-1 and BCL-2 (Fig. 6b) and memory subset distribution with transitional and effector memory subsets being dominant (Supplementary Fig. 9b). Again, the frequencies of the BCL-2^hi^ memory pool within the spike-specific CD8^+^ T cells were stable before and after the 4^th^ antigen contact irrespective of breakthrough infection or 4^th^ vaccine dose (Fig. 6c). Hence, vaccine-elicited spike-specific CD8^+^ T cell immunity exhibited a substantial recall capacity in vivo even towards VOCs such as Omicron. However, the neutralizing antibody response after the 4^th^ antigen contact differed from the spike-specific CD8^+^ T cells in the following aspects (Fig. 6d): First, neutralization capacity depended on the targeted SARS-CoV-2 variant regardless of whether the 4^th^ antigen exposure was through vaccination or breakthrough infection. Second, the neutralizing antibody response depended on the 4^th^ antigen contact with differences in vaccination and breakthrough infections. Third, the neutralizing antibody response exhibited a prolonged decay after breakthrough infections compared to vaccination. Thus, in contrast to spike-specific CD8^+^ T cells, recall of the neutralizing antibody response was less robust with respect to VOCs.

## Discussion

Here, we mapped the dynamics of mRNA vaccine- versus SARS-CoV-2 infection-boosted spike-specific adaptive effector immunity. By analyzing the dominant A*01/S_865_- and A*02/S_269_-specific CD8^+^ T cell responses, we observed a rapid and robust expansion of spike-specific CD8^+^ T cells with a similar slope and amplitude after the 3^rd^ and 4^th^ vaccine dose versus breakthrough infections with SARS-CoV-2 Delta and Omicron variants. Notably, kinetics and magnitude were comparable to the spike-specific CD8^+^ T cell response after the 2^nd^ vaccine dose with a remarkably high recruitment of spike-specific CD8^+^ T cells to the effector pool that is reflected by high expression of activation and proliferation markers, like CD38 and Ki-67. This observation highlights the effectiveness of mRNA vaccination to elicit rapid and functional CD8^+^ T cell responses. Thus, given the important role of CD8^+^ T cells to control SARS-CoV-2 infection^14–17^, mRNA booster vaccination represents a valuable tool to immediately react to high viral burden in the population in order to e.g., protect vulnerable groups and to reduce the risk of overwhelming the public health system.

Importantly, however, the spike-specific CD8^+^ T effector cell response after the 3^rd^ mRNA vaccination only lasted approx. 1-2 months until it decreased to a similar level as prior booster vaccination. This rapid contraction of the spike-specific CD8^+^ T cell response should be taken into account when booster vaccination strategies are planned especially for risk groups but also for broader populations. Furthermore, the steep decline of the spike-specific CD8^+^ T cell boost response is in contrast to a more prolonged contraction reported for non-spike epitope-specific CD8^+^ T cells after SARS-CoV-2 infection^11^. This may indicate a difference in virus-specific CD8^+^ T cell kinetics induced by SARS-CoV-2 infection versus mRNA vaccination. Possible explanations include differences in antigen half-life, innate immunity, co-stimulation, cytokines, antigen presentation and CD4^+^ T cell responses that are all required for coordinating CD8^+^ T cell responses^4,15,16^ after mRNA vaccination versus SARS-CoV-2 infection.

A durable and functionally competent spike-specific CD8^+^ T memory cell response was already detectable after the 2^nd^ dose of mRNA vaccine and thus after completed basic immunization^3,4,18^. Here, we now show that this well-established durable spike-specific CD8^+^ T memory cell response is not tremendously affected by the 3^rd^ dose. In particular, a pool of T_SCM_ cells that have been reported to maintain long-term CD8^+^ T cell immunity after yellow fever virus (YFV) vaccination^12,19^ and have been shown to be detectable after basic immunization with SARS-CoV-2 mRNA vaccination^20^ were already induced within spike-specific CD8^+^ T cells after prime vaccination. The spike-specific CD8^+^ T_SCM_ cell pool reached final size after the 2^nd^ dose and remained constant throughout the 3^rd^ and 4^th^ dose. Thus, 3^rd^ mRNA vaccination does neither amplify long-term CD8^+^ T cell immunity nor drive the CD8^+^ T cell memory pool into relevant senescence. This is in line with a recent report showing that repeated antigen exposure does also not induce T cell exhaustion of spike-specific CD8^+^ T cells^21^. It rather appears that the spike-specific CD8^+^ T cell booster response is an effector response based on a stable memory pool. Of note, spike-specific CD8^+^ T cell epitope spreading after the 3^rd^ mRNA vaccination is also not detectable^10^ further supporting the stability of the memory pool. Importantly, spike-specific CD8^+^ T memory cells were capable to robustly mount recall responses in vitro and in vivo after breakthrough infections with Delta or Omicron. This recall robustness is explained by the previously described cross-reactivity of the vaccine-elicited spike-specific CD8^+^ T cell response^10,18,22–25^, diversification of the T cell repertoire^21^ and of the rapid kinetics and effector differentiation dynamics shown in this study. It is, however, important to note that our study is limited to a small cohort of healthy, rather young individuals without higher risk to develop severe COVID-19^16^ and with the capability to establish robust vaccine-elicited immune responses^2–5,18^. Future studies have to show whether individuals above the age of 60 develop comparable immunity after mRNA vaccination. Still, our observations challenge the discussion about the necessity to frequently apply mRNA booster vaccination in three-to-six-months intervals to healthy individuals that are not compromised in their immune response but have also been included in the large vaccination campaigns thus far.

In contrast to spike-specific CD8^+^ T cells, boosting the neutralizing antibody response by currently applied, non-adapted mRNA vaccines clearly depends on the targeted SARS-CoV-2 variant. In particular, neutralizing capacity against Omicron is hardly increased. Of note, neutralizing capacity against Omicron is also only moderately increased after breakthrough infections with Omicron probably reflecting antibody escape. Similar to the spike-specific CD8^+^ T cell response, the neutralizing capacity declines slower after infection versus vaccination. Yet, it appears that neither after vaccination nor after infection the neutralization capacity of the humoral response is sufficient to protect from Omicron infection as indicated by the high numbers of breakthrough infections in vaccinees and convalescents. In sum, our study highlights that mRNA vaccines are potent inducers of a robust, functionally competent and durable spike-specific CD8^+^ T cell immunity already after completed basic immunization. This has also important implications for vaccine development targeting other CD8^+^ T cell-controlled infectious agents and cancers.

## Methods

### Study cohort

In total, 38 individuals receiving SARS-CoV-2 vaccinations were recruited at the Freiburg University Medical Center, Germany. Of those, blood was collected from 31 individuals vaccinated three times with the mRNA vaccines bnt162b/Comirnaty or mRNA-1273/Spikevax and 5 individuals receiving a 4^th^ vaccination. All vaccinees did not have a history of SARS-CoV-2 infection prior to inclusion confirmed by seronegativity for anti-SARS-CoV-2 nucleocapside IgG (anti-SARS-CoV-2 N IgG). Moreover, blood was collected from 13 individuals with SARS-CoV-2 breakthrough infections after a 3^rd^ mRNA vaccination. Breakthrough infections were confirmed by positive PCR-testing from oropharyngeal swab. All 13 individuals with breakthrough infections included in this study had mild symptoms without respiratory insufficiency (according to WHO guidelines^26^). Characteristics of the participants are summarized in Supplementary Table 1, including the results of the HLA-genotyping performed by next-generation sequencing.

### Ethics

Written informed consent was obtained from all study participants. The study was conducted in accordance to federal guidelines, local ethics committee regulations (Albert-Ludwigs-Universität, Freiburg, Germany; vote: 322/20, 21-1135 and 315/20) and the Declaration of Helsinki (1975).

### PBMC isolation

PBMCs were isolated from venous blood samples collected in EDTA blood collection tubes by density centrifugation with lymphocyte separation medium (Pancoll separation medium, PAN Biotech GmbH). PBMCs were stored at −80 °C until further processing. The cells were thawed in prewarmed RPMI cell culture medium supplemented with 10% fetal calf serum, 1% penicillin/streptomycin, 1.5% 1 M HEPES (all purchased from Thermo Scientific) and 50 U/mL Benzonase (Sigma).

### Sequence alignment

Sequence homology was analyzed in Geneious version 11.0.5 (https://www.geneious.com/) using Clustal Omega version 1.2.2 alignment with default settings^27^. Reference genome of human ancestral SARS-CoV-2 (MN908947.3) was obtained from NCBI database. Genome sequences of SARS-CoV-2 variants of concern (VOCs) B.1, B.1.1.7, B.1.351, P.1, B.1.617.2, B.1.1.529 BA.1 and B.1.1.529 BA.2 were identified via CoVariants (https://covariants.org/). Spike epitopes in ancestral strain and all VOCs were aligned according to their homology on an amino acid level.

### Peptides and tetramers for T cell analysis

Peptides were manufactured with an unmodified N-terminus and an amidated C-terminus with standard Fmoc chemistry (Genaxxon Bioscience). All peptides showed a purity of >70%. To generate tetramers, SARS-CoV-2 spike peptides (A*01/S_865_: LTDEMIAQY, A*02/S_269_: YLQPRTFLL) were loaded on biotinylated HLA class I (HLA-I) easYmer (immunAware) according to manufacturer’s instructions. Subsequently, peptide-loaded-HLA class I monomers were tetramerized with phycoerythrin (PE)-conjugated streptavidin according to the manufacturer’s instructions.

### In vitro expansion of spike-specific CD8^+^ T cells and assessment of effector function

1.5 × 10^6^ PBMCs were stimulated with the spike protein-derived peptides A*01/S_865_ or A*02/S_269_ and anti-CD28 monoclonal antibody (0.5 µg/mL) for 14 days in RPMI cell culture medium supplemented with rIL-2 (20 IU/ml, StemCell Technologies). At day 4, 7 and 11, 50% of the culture medium was exchanged with freshly prepared medium containing 20 IU/mL rIL-2. After 14 days, PBMCs were stimulated with peptides again, and stained for CD107a for 1 h at 37 °C to analyze degranulation. Subsequently, brefeldin A (GolgiPlug, 0.5 μl/mL) and monensin (GolgiStop, 0.5 μl/mL) (all BD Biosciences) were added and incubation continued for four more hours, followed by surface and intracellular staining with anti-IFNy, anti-TNF and anti-IL-2-specific antibodies. For calculation of the expansion capacity and to assess the cytotoxic capacity of the expanded cells, peptide-loaded HLA class I tetramer staining was performed together with intracellular staining of Granzyme B, Granzyme K, Perforin and Granulysin.

### Magnetic bead-based enrichment of spike-specific CD8^+^ T cells

CD8^+^ T cells targeting spike epitopes were enriched as described previously^28^. In brief, 5 × 10^6^ to 20 × 10^6^ PBMCs were stained with PE-coupled peptide-loaded HLA class I tetramers for 30 min at room temperature followed by incubation with magnetic anti-PE microbeads. Subsequent positive selection of magnetically labelled cells was achieved by using MACS technology (Miltenyi Biotec) according to the manufacturer’s protocol. The enriched spike-specific CD8^+^ T cells were analyzed using multicolor flow cytometry. Cell frequencies were calculated as previously described^28^. Of note, only samples with ≥ 5 non-naïve spike-specific CD8^+^ T cells were included in subsequent analyses. Accordingly, the detection limit of spike-specific CD8^+^ T cells in this study was 0.25 – 1 × 10−^6^, depending on the initial cell input. This cut-off number has been applied and validated in different studies on antigen-specific T cells and has shown to generate reproducible results^3,11,29,30^.

### Multiparametric flow cytometry for T cell analysis

Antibodies used for multiparametric flow cytometry are listed in Supplementary Table 2. To facilitate staining of intranuclear and cytoplasmic targets, FoxP3/Transcription Factor Staining Buffer Set (Thermo Fisher) and Fixation/Permeabilization Solution Kit (BD Biosciences) were used, respectively. Finally, cells were fixed in 2% paraformaldehyde (Sigma) and samples were analyzed on FACSCanto II or LSRFortessa with FACSDiva software version 10.6.2 (BD), or CytoFLEX (Beckman Coulter) with CytExpert Software version 2.3.0.84. Further analyses of the data were performed using FlowJo version 10.6.2 (Treestar). Phenotypical analyses were based on 5 ×10^6^ to 20 ×10^6^ PBMCs that were used as an input number for the magnetic bead-based enrichment of spike-specific CD8^+^ T cells.

### Dimensional reduction of multiparametric flow cytometry data

For dimensionality reduction, flow cytometry data were analyzed with R version 4.1.1 and the Bioconductor CATALYST package (release 3.13)^31^. Initially, viable and tetramer-positive CD8^+^ T cells (or subsets of those) were identified using FlowJo 10 in two separate multiparametric flow cytometry panels (activation panel: HLA-DR, BCL-2, PD-1, CD137, Ki67, TCF-1, EOMES, T-BET, TOX, CD38, CD45RA, CCR7; differentiation panel: CD45RA, CCR7, CD27, CD28, CD127, CD11a, CD57, CXCR3, CD95, CD57, CD39, KLRG1, PD-1). To facilitate visualization of the dimensionality reduction by *t*-SNE and diffusion map analysis, cell counts were sampled down to at least 20 cells per sample, and marker expression intensities were transformed by arcsinh-transformation with a cofactor of 150.

### Serum IgG determination

Determination of SARS-CoV-2-specific antibodies was performed by using the Euroimmun assay ‘Anti-SARS-CoV-2-QuantiVac-ELISA (IgG)’ for detecting anti-SARS-CoV-2 spike IgG (anti-SARS-CoV-2 S IgG; <35.2 BAU/mL: negative, ≥ 35.2 BAU/mL: positive) and the Mikrogen assay ‘recomWell SARS-CoV-2 (IgG)’ for detecting anti-SARS-CoV-2 N IgG (detection limit, 24 a.u. ml^−1^) according to the manufacturer’s instructions. Data were collected with the SparkControl Magellan software version 2.2.

### Neutralization assay

Samples of vaccinated individuals and those with breakthrough infections were tested in a plaque reduction neutralization assay as previously described^3^. In brief, VeroE6 cells were seeded in 12-well plates at a density of 4 × 10^5^ cells per well. Serum samples were diluted at ratios of 1:16, 1:32, 1:64, 1:128, 1:256, 1:512 and 1:1024 in a total volume of 50 μl PBS. For each sample, a serum-free negative control was included. Diluted sera and negative controls were subsequently mixed with 90 plaque-forming units (PFU) of authentic SARS-CoV-2 (either B.1, B.1.617.2 (delta) and B.1.1.529 BA.1 (omicron)) in 50 μl PBS (1,600 PFU/mL) resulting in final sera dilution ratios of 1:32, 1:64, 1:128, 1:256, 1:512, 1:1024 and 1:2048. After incubation at room temperature for 1 h, 400 μl PBS was added to each sample and the mixture was subsequently used to infect VeroE6 cells 24 h after seeding. After 1.5 h of incubation at room temperature, inoculum was removed and the cells were overlaid with 0.6% Oxoid-agar in DMEM, 20 mM HEPES (pH 7.4), 0.1% NaHCO3, 1% BSA and 0.01% DEAE-Dextran. Cells were fixed 72 h after infection using 4% formaldehyde for 30 min and stained with 1% crystal violet upon removal of the agar overlay. PFU were counted manually. Plaques counted for serum-treated wells were compared to the average number of plaques in the untreated negative controls, which were set to 100%. Calculation of PRNT50 values was performed using a linear regression model in GraphPad Prism 9 (GraphPad Prism Software).

### Statistics

GraphPad Prism software version 9.3.1 was used for statistical analysis. Statistical significance was assessed by Kruskal-Wallis test, one-way ANOVA with mixed-effects model, two-way ANOVA with full model and main model. Statistical analysis was performed for A*01/S_865_ (*n* = 7) and A*02/S_269_ (*n* = 8) longitudinally analyzed CD8^+^ T cell responses in Figs. 1a, b, 3c, 4a, b and Supplementary Figs. 2a, 5a–c, 7c–e for *n* = 28 subjects longitudinally followed in Fig. 2a, for A*01/S_865_ (*n* = 2) and A*02/S_269_ (*n* = 3) T cell responses longitudinally followed in Fig. 2c, for *n* = 26 subjects in Fig. 2b, for *n* = 6 prepandemic samples Supplementary Fig. 1c, for *n* = 2 subjects in Supplementary Fig. 3c, for *n* = 7 at 3 months after 2^nd^ vaccination, *n* = 11 at 9 months after 2^nd^ vaccination and *n* = 11 at 3 months after 3^rd^ vaccination in Fig. 3a and Supplementary Fig. 4b, for *n* = 4 at 3 months after 2^nd^ vaccination, *n* = 8 at 9 months after 2^nd^ vaccination and *n* = 10 at 3 months after 3^rd^ vaccination in Supplementary Fig. 4b, for A*01/S_865_ (*n* = 7) and A*02/S_269_ (*n* = 6) longitudinally analyzed CD8^+^ T cell responses in Fig. 3d, for *n* = 8 at 3 months after 2^nd^ vaccination, *n* = 12 at 9 months after 2^nd^ vaccination and *n* = 11 at 3 months after 3^rd^ vaccination in Fig. 3b, for *n* = 4 in Supplementary Fig. 6a, for A*01/S_865_ (*n* = 2) and A*02/S_269_ (*n* = 2) longitudinally analyzed CD8^+^ T cell responses in Supplementary Fig. 6b, for *n* = 10 at 3 months after 2^nd^ vaccination, *n* = 12 at 9 months after 2^nd^ vaccination and *n* = 11 at 3 months after 3^rd^ vaccination in Fig. 4c, for *n* = 10 at 3 months after 2^nd^ vaccination, *n* = 11 at 9 months after 2^nd^ vaccination and *n* = 11 at 3 months after 3^rd^ vaccination in Fig. 4d, for *n* = 6 at 3 months after 2^nd^ vaccination, *n* = 12 at 9 months after 2^nd^ vaccination and *n* = 10 at 3 months after 3^rd^ vaccination in Fig. 4e, for *n* = 6 at 3 months after 2^nd^ vaccination, *n* = 12 at 9 months after 2^nd^ vaccination and *n* = 11 at 3 months after 3^rd^ vaccination in Fig. 4f, for Omicron infection *n* = 12, Delta infection *n* = 2 and 4^th^ vaccination *n* = 5 longitudinally analyzed T-cell responses in Fig. 5a, for Omicron infection *n* = 11, Delta infection n = 2 and 4^th^ vaccination *n* = 4 analyzed T cell responses in Fig. 5b and in peak response in Supplementary Fig. 8a, for Omicron infection *n* = 12, Delta infection *n* = 2 and 4^th^ vaccination *n* = 3 longitudinally analyzed T cell responses in Fig. 6c, for Omicron infection *n* = 11, Delta infection *n* = 1 and 4^th^ vaccination *n* = 3 in Fig. 6d, for Omicron infection *n* = 6, Delta infection *n* = 2 and 4^th^ vaccination *n* = 4 analyzed T cell responses after 1 month in Supplementary Fig. 8a and Supplementary Fig. 9b, for Omicron infection *n* = 6, Delta infection *n* = 2 and 4^th^ vaccination *n* = 4 analyzed T cell responses in Supplementary Fig. 9a.

### Reporting summary

Further information on research design is available in the Nature Research Reporting Summary linked to this article.

## Supplementary information

Supplementary Information

Peer Review File

Reporting Summary

## Data Availability

The raw values for charts and graphs are available in the Source Data file whenever possible. All requests for additional raw (especially flow cytometry data) and materials are promptly reviewed by the University of Freiburg Center for Technology Transfer to verify if the request is subject to any intellectual property or confidentiality obligations. Donor-related data not included in the paper were generated as part of clinical examination and may be subject to donor confidentiality. Any data and materials that can be shared will be released via a Material Transfer Agreement. Source data are provided with this paper.

## Source data

Source Data

## Code availability

R code to reproduce the analyses of multiparametric flow-cytometry data is available at https://github.com/sagar161286/SARSCoV2_specific_CD8_Tcells.
