# Supplementary material for: COVID-19 mRNA booster vaccine induces transient CD8+ T effector cell responses while conserving the memory pool for subsequent reactivation
Source: Nat Commun. 2022 Aug 8;13:4631. doi: 10.1038/s41467-022-32324-x (PMC9358914; doi:10.1038/s41467-022-32324-x)
Supplement: Supplementary file 1 — Supplementary Information [file 41467_2022_32324_MOESM1_ESM.pdf]

Supplementary Figure 1

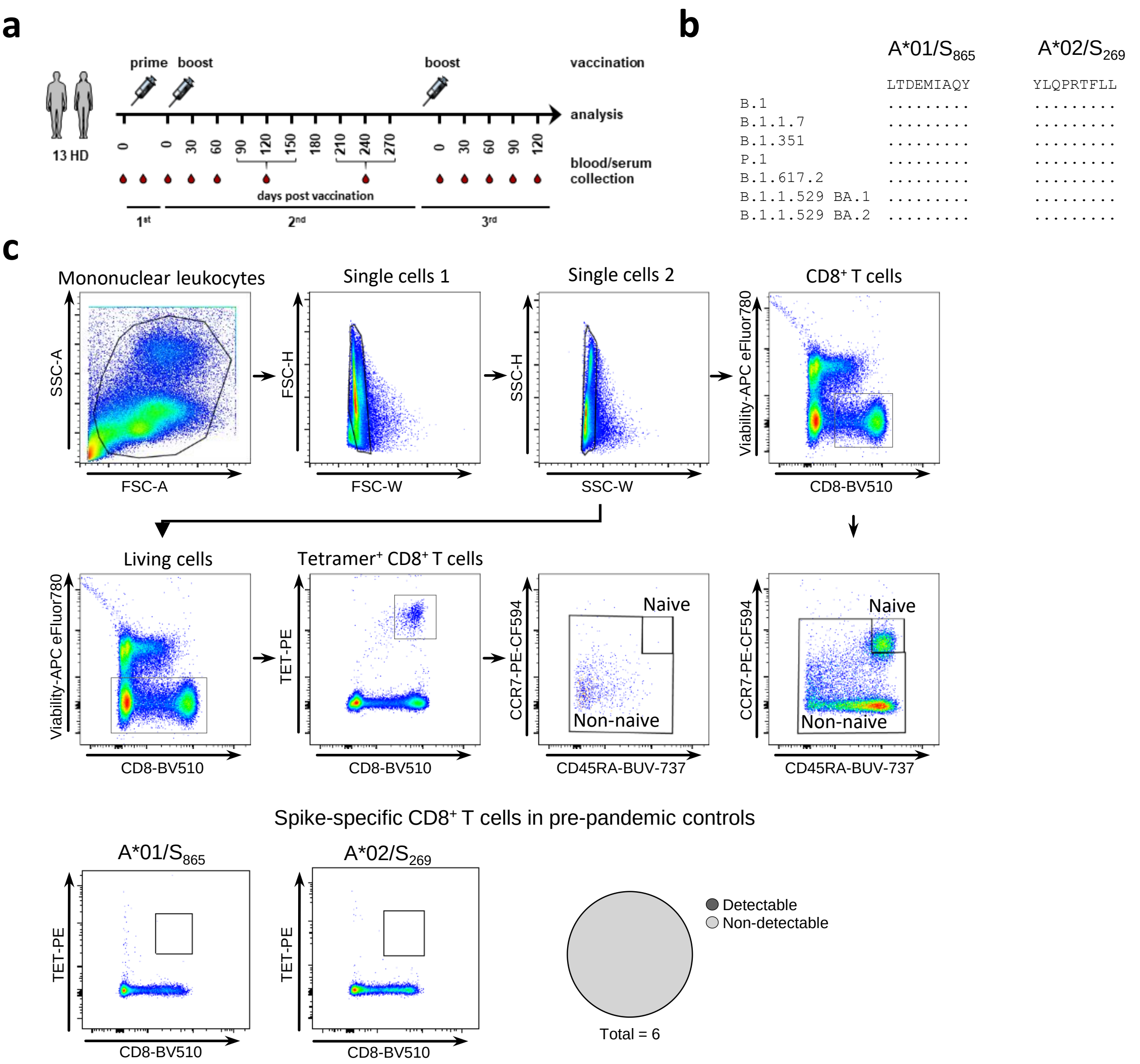

Study design and gating strategy

Vaccination and blood donation schedule. Graphic was created by using pictures from Servier Medical Art (http://smart.servier.com/) and licensed under a Creative Common Attribution 3.0 Generic License (**a**). Epitope sequences for A*01/S_{865}- and A*02/S_{269}-specific CD8$^+$ T cell epitopes with respect to current circulating variants of concern (**b**). Gating strategy of flow cytometry data to specify living, single cell, non-naïve, virus-specific CD8$^+$ T cells and representative dot plots of pre-pandemic control samples after tetramer-based enrichment (**c**). This gating was applied for all samples to identify spike-specific CD8$^+$ T cells.

Supplementary Figure 2

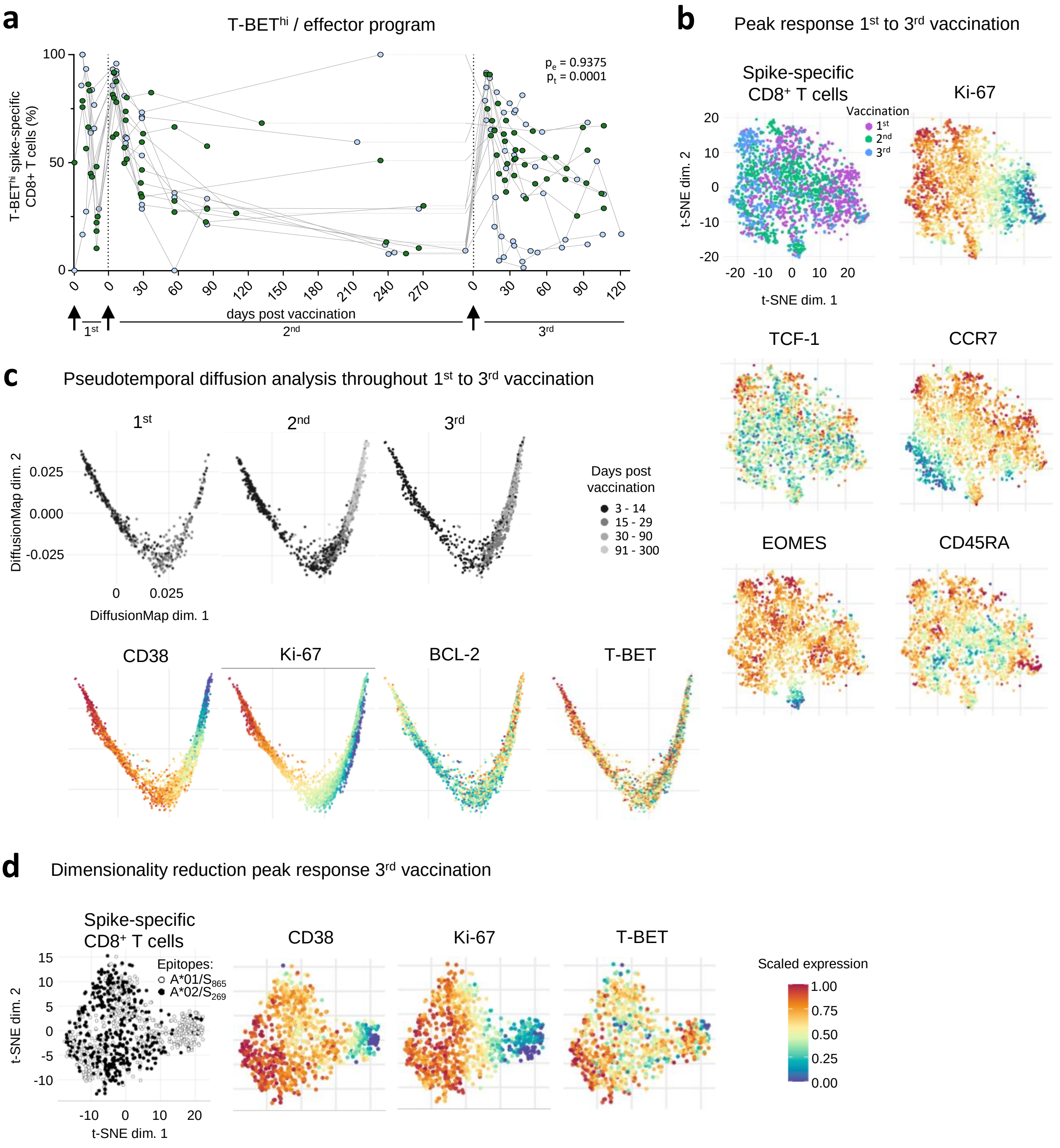

Characterization of the effector response after the 1st, 2nd and 3rd vaccine dose

T-BET expression within spike-specific non-naïve CD8+ T cells (**a**). t-SNE representation of flow cytometry data comparing spike-specific $CD8^+$ T cells at peak response after 1st, 2nd and 3rd vaccination (**b**). Expression levels of Ki-67, TCF-1, CCR7, EOMES and CD45RA are depicted. Diffusion map analysis of flow cytometry data for spike-specific $CD8^+$ T cells throughout 1st, 2nd and 3rd vaccination (**c**). Expression levels of CD38, Ki-67, BCL-2 and T-BET are depicted. t-SNE representation of flow cytometry data comparing A*01/S_{865}- and A*02/S_{269}-specific $CD8^+$ T cells at peak response after 3rd vaccination (**d**). Expression levels of CD38, Ki-67 and T-BET are depicted. Statistical significance was calculated by two-way ANOVA with main model (a) to compare the effects of targeted epitopes (p_e) and time course (p_t). Source data are provided as a Source Data file.

Supplementary Figure 3

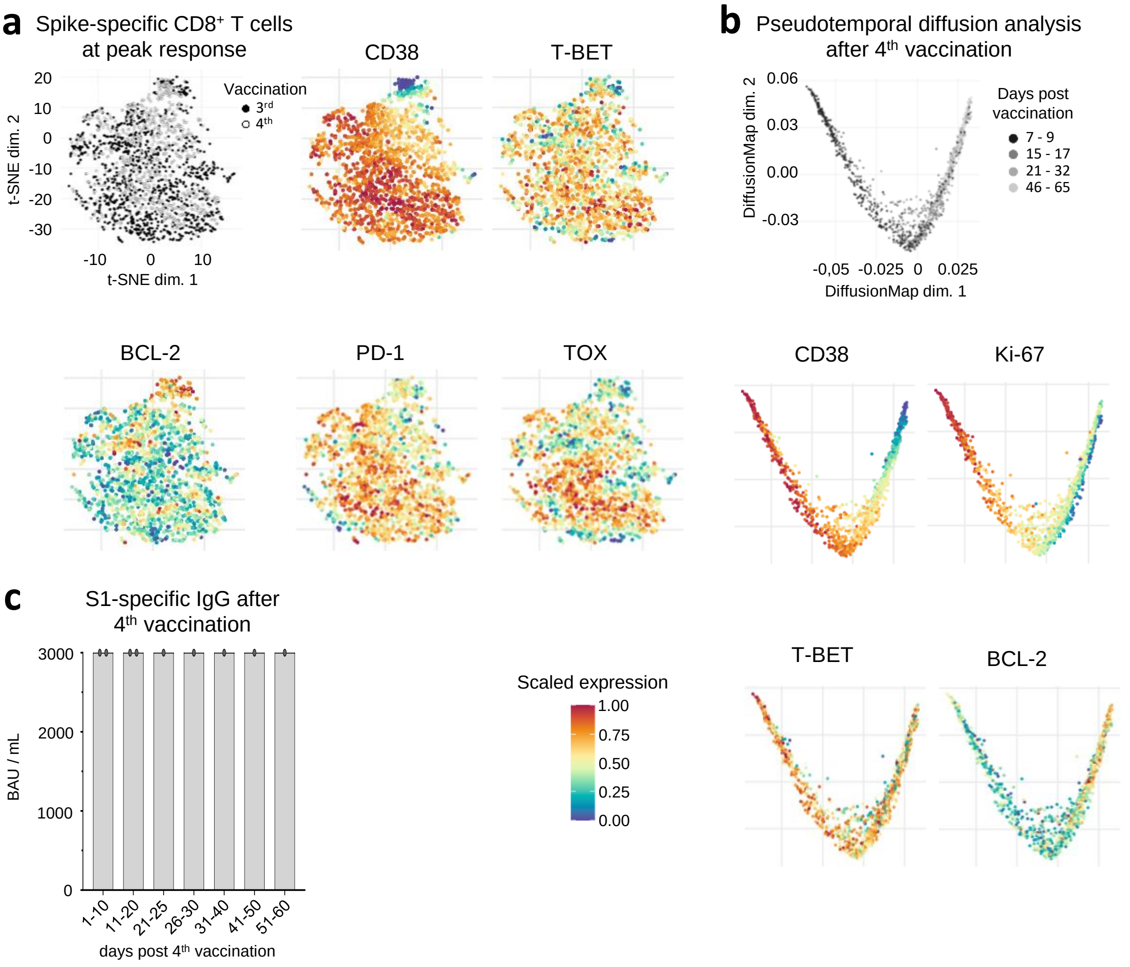

Effector response after the 4th vaccine dose

t-SNE representation of flow cytometry data at peak response after 3rd and 4th vaccination (**a**). Expression levels of CD38, T-BET, BCL-2, PD-1 and TOX are depicted. Pseudotemporal diffusion map analysis of flow cytometry data for spike-specific CD8+ T cells after 4th vaccination (**b**). Expression levels of CD38, Ki-67, T-BET and BCL-2 are depicted. Quantification of serum anti-SARS-CoV-2 spike IgG levels following 4th vaccination with n=2 individuals tested (**c**). Median values are depicted with 95% confidence interval error bars. Statistical significance was not calculated due to limited sample sizes. Source data are provided as a Source Data file.

Supplementary Figure 4

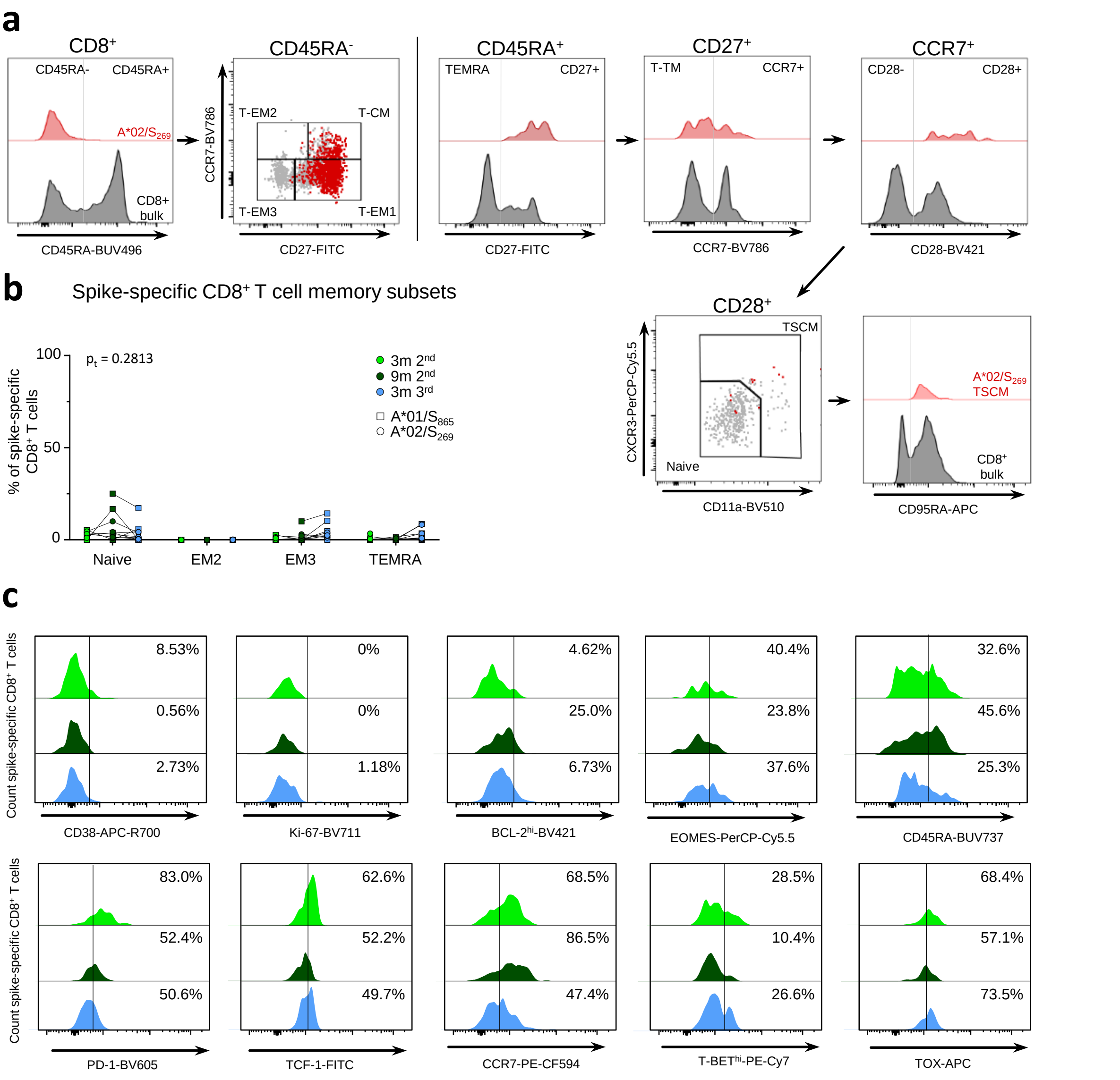

Spike-specific CD8+ T memory cells

Gating strategy of flow cytometry data for memory subset specifications (**a**). This gating was applied to samples after *ex vivo* enrichment. Composition of spike-specific CD8+ T cell subsets at 3 (n=7) and 9 (n=11) months after 2nd, and 3 (n=11) months after 3rd vaccination (**b**). Representative histograms for indicated molecule expression at 3 and 9 months post 2nd, and 3 months post 3rd vaccination (**c**). Tukey's test was applied for multiple comparisons to examine the effect of sampling time points (p_t) on memory subsets and marker expression (b). Source data are provided as a Source Data file.

Supplementary Figure 5

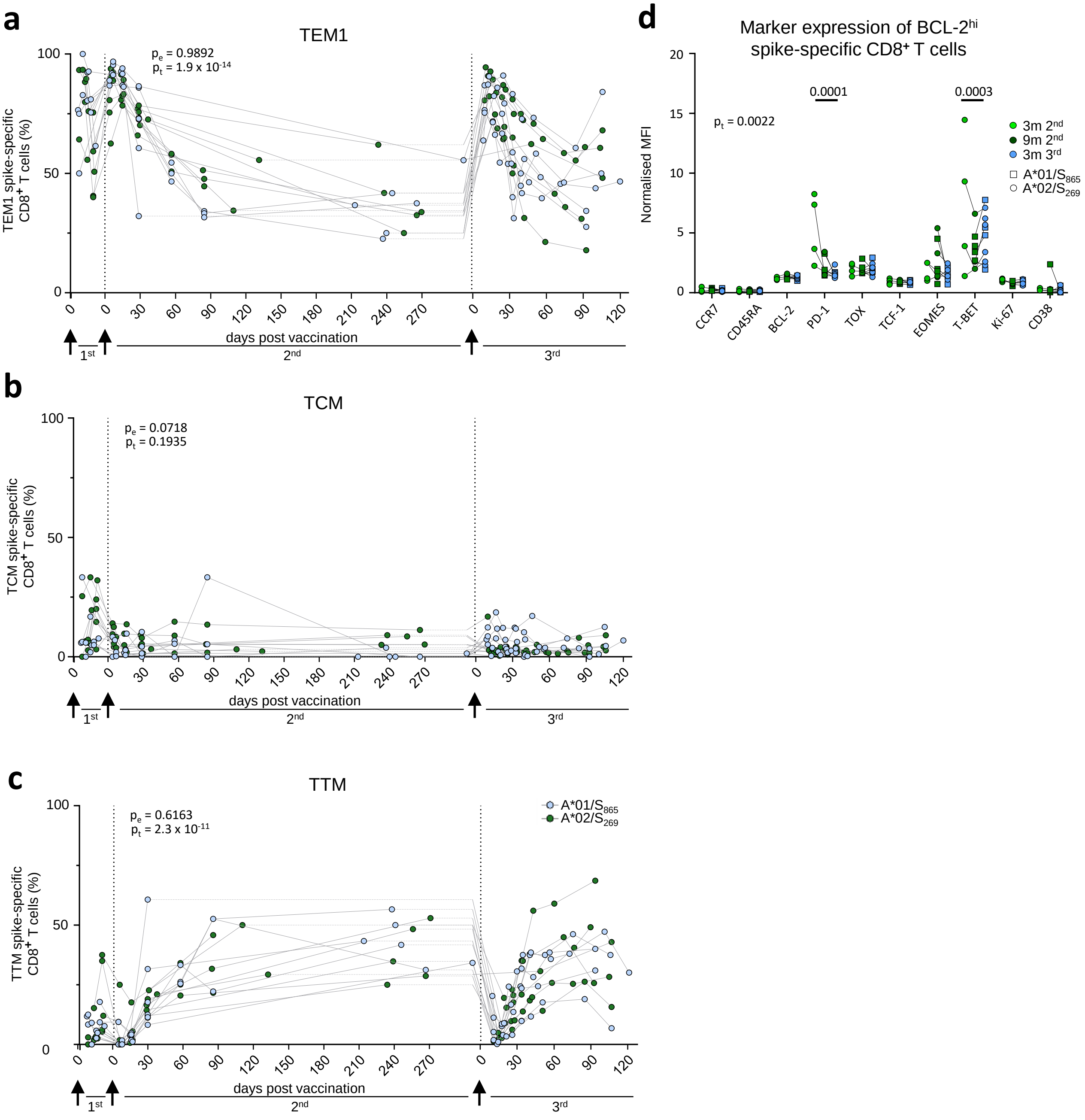

Longitudinal characterization of spike-specific CD8⁺ T memory cells

Proportion of TEM1 (**a**), TCM (**b**) and TTM (**c**) subsets of spike-specific CD8⁺ T cells throughout 1st to 3rd vaccination. Marker expression of BCL-2hi non-naïve spike-specific CD8⁺ T cells at 3 (n=4) and 9 (n=8) months post 2nd, and 3 (n=10) months post 3rd vaccination normalized to bulk naive CD8⁺ T cells (**d**). Statistical significance was calculated by two-way ANOVA with full model (a, b, c) and Tukey's test for multiple comparisons (d) to examine the effect of targeted epitopes (p_e; a, b, c) and of sampling time points (p_t; a, b, c, d) on memory subsets and marker expression. Source data are provided as a Source Data file.

Supplementary Figure 6

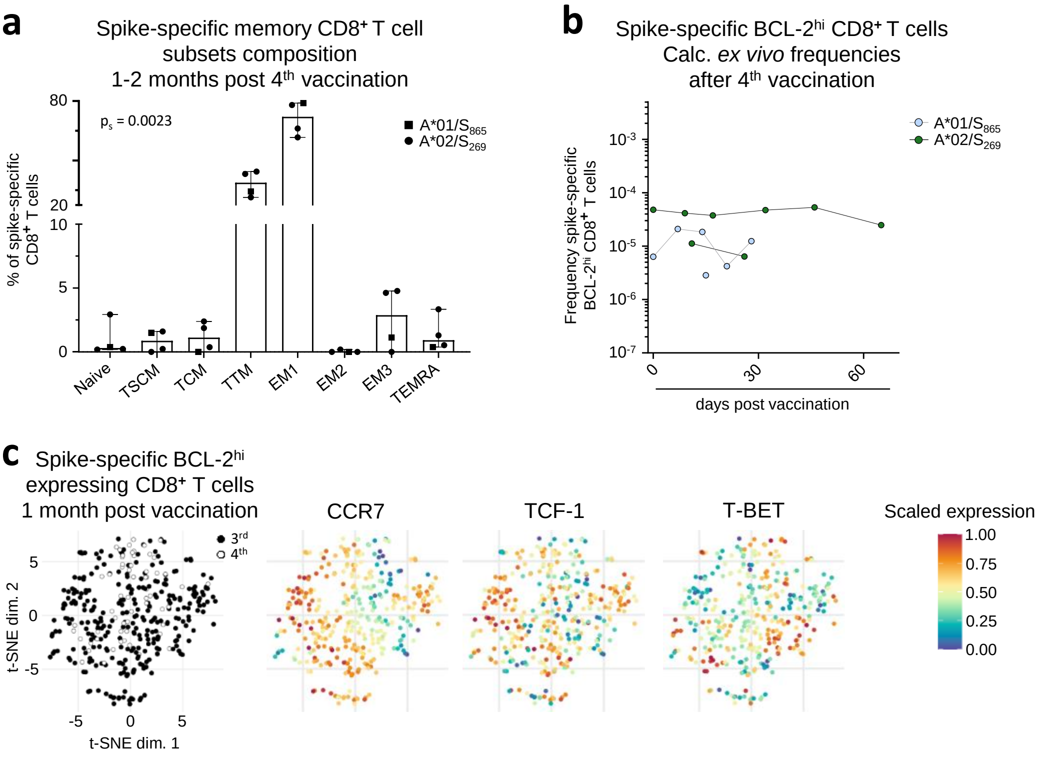

Spike-specific CD8⁺ T memory cells after the 4th vaccine dose

Composition of spike-specific CD8⁺ T cell subsets 1-2 months after 4th vaccination with n=4 individuals tested (**a**). Median values are depicted with 95% confidence interval error bars. Calculated *ex vivo* frequencies of BCL-2hi non-naive spike-specific CD8⁺ T cells after 4th vaccination during the first 70 days after vaccination (**b**). t-SNE representation comparing BCL-2hi expressing spike-specific CD8⁺ T cells 1 month after 3rd and 4th vaccination (**c**). Expression levels of CCR7, TCF-1 and T-BET are depicted for all analyzed cells together. Statistical significance was calculated by Kruskal-Wallis test (a) for memory subset composition. Source data are provided as a Source Data file.

Supplementary Figure 7

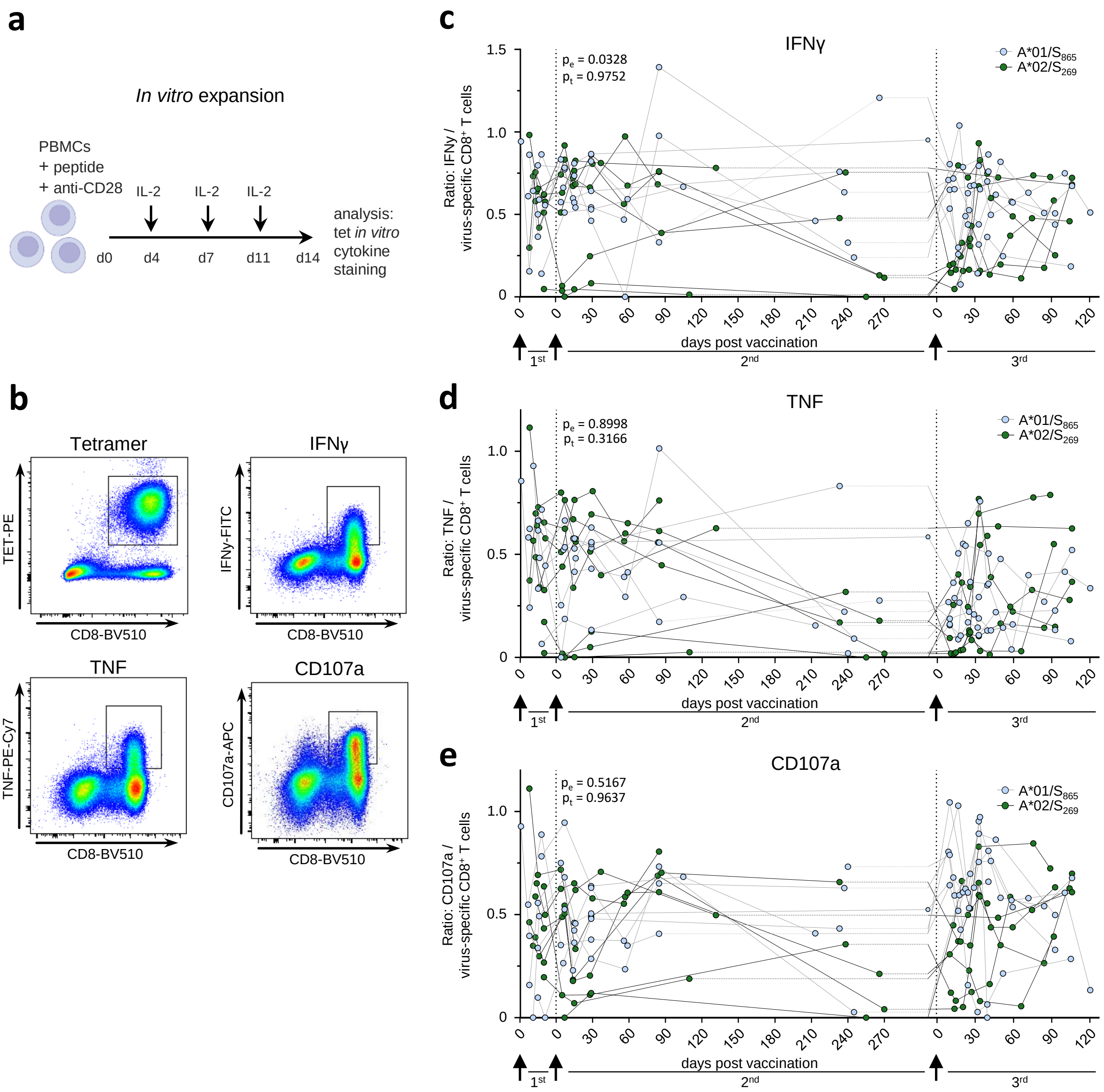

Reactivation capacity of spike-specific CD8+ T cells throughout the 1st to 3rd vaccine dose

Experimental design of peptide-specific *in vitro* expansion of CD8+ T cells. Graphical items are created with Biorender.com (**a**). Dot plots showing spike-specific CD8+ T cells, and IFNγ-, TNF- and CD107a production of expanded CD8+ T cells after peptide stimulation (**b**). Percentage of CD8+ T cells expressing IFNγ **(c)**, TNF **(d)** and CD107a **(e)** upon peptide stimulation related to the percentage of spike-specific CD8+ T cells from all CD8+ T cells throughout 1st, 2nd and 3rd vaccination after *in vitro* expansion. Statistical significance was calculated by two-way ANOVA with main model (c, d, e) to compare the effects of targeted epitopes (p_e) and time course (p_t). Source data are provided as a Source Data file.

Supplementary Figure 8

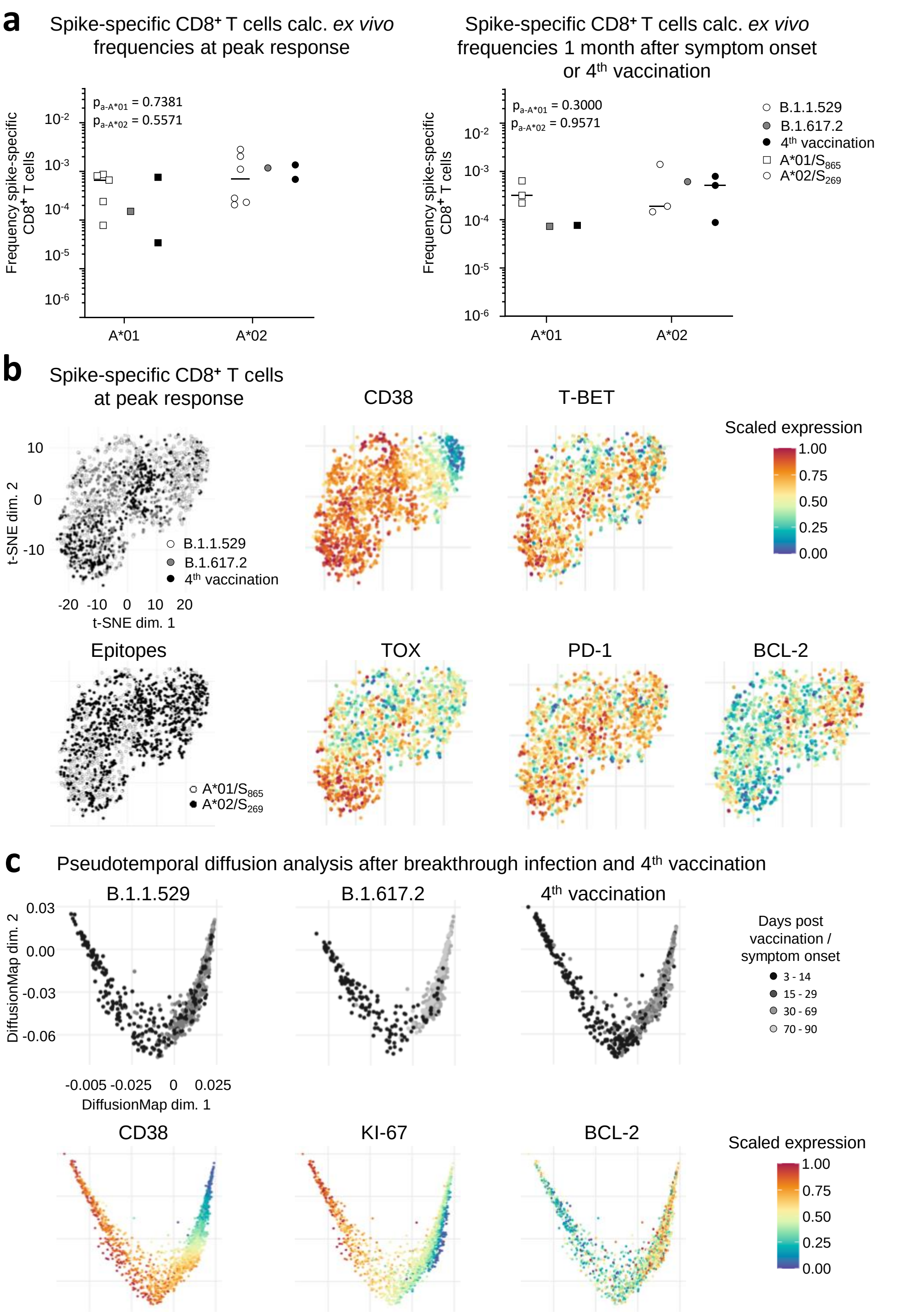

Characterization of CD8⁺ T effector cell response after breakthrough infection and the 4th vaccine dose

Calculated *ex vivo* frequencies of spike specific CD8⁺ T cells after breakthrough infections and 4th vaccination at peak response (n=2 for Delta, n=11 for Omicron and n=4 for 4th vaccination) and 1 month after symptom onset or 4th vaccination (n=2 for Delta, n=6 for Omicron and n=4 for 4th vaccination) (**a**). t-SNE representation of spike-specific CD8⁺ T cells at peak response after breakthrough infection and 4th vaccination. Expression levels of CD38, T-BET, TOX, PD-1 and BCL-2 are depicted (**b**). Pseudotemporal diffusion map analysis of spike-specific CD8⁺ T cells after symptom onset and 4th vaccination (**c**). Expression levels of CD38, Ki-67, BCL-2 are depicted. Statistical significance was calculated by Kruskal-Wallis test (a) to compare the effects of antigen triggers (p_a) on epitope-specific T cell frequencies. Source data are provided as a Source Data file.

Supplementary Figure 9

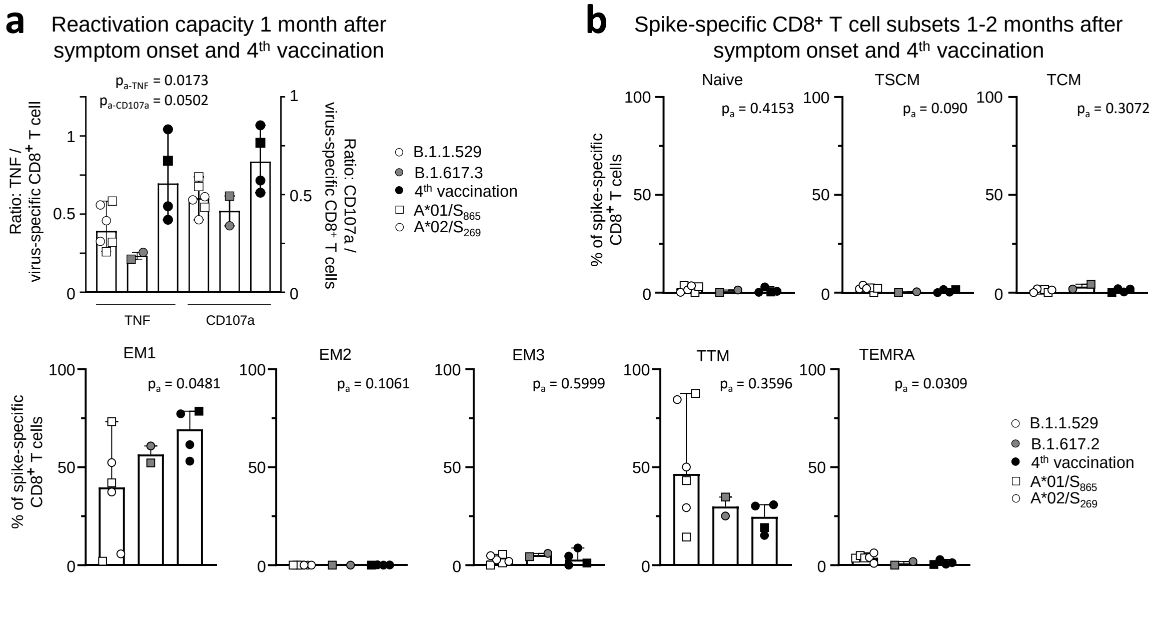

Spike-specific CD8+ T memory cells after breakthrough infection and the 4th vaccine dose

Reactivation capacity of spike-specific CD8+ T cells 1 month after symptom onset and 4th vaccination with n=6 for Omicron and n=2 for Delta breakthrough infections, and n=4 individuals receiving a 4th vaccination (**a**). Composition of spike-specific CD8+ T cell subsets 1 month after breakthrough infection and 4th vaccination with n=6 for Omicron and n=2 for Delta breakthrough infections, and n=4 individuals receiving a 4th vaccination (**b**). Median values are depicted with 95% confidence interval error bars (a, b). Statistical significance was calculated by Kruskal-Wallis test (a, b) to compare the effects of antigen triggers (p_a) on T cell reactivation capacity (a) and memory subset distribution (b). Source data are provided as a Source Data file.

Supplementary Table 1: Donor characteristics

Donor ID	Sex	Age	HLA Type	Cohort	T cell assay	Tested Epitopes	Serum S-IgG1 assay	Serum N-IgG assay	Neutralizing Antibodies (Plaque reduction assay)
V1	f	46	A*02:01, A*33:03, B*44:03, B*58:01	vaccinated	yes	A*02/S269-277	no	yes	no
V2	f	32	A*01:01, A*02:01, B*07:02, B*37:01	vaccinated	yes	A*01/S865-873<br>A*02/S269-277	yes	yes	yes
V3	f	45	A*01:01, A*11:01, B*15:17, B*35:01	vaccinated	yes	A*01/S865-873	yes	yes	yes
V4	f	27	A*01:01, A*32:01, B*44:02, B*52:01	vaccinated	yes	A*01/S865-873	no	yes	no
V5	f	61	A*02:01, B*07:02, B*44:02	vaccinated	yes	A*02/S269-277	yes	yes	yes
V6	m	32	A*02:01, B*15:01, B*51:01	vaccinated	yes	A*02/S269-277	yes	yes	yes
V7	f	52	A*02:01, A*68:01, B*15:01, B*44:02	vaccinated	yes	A*02/S269-277	yes	yes	yes
V8	m	36	A*02:01, A*68:01, B*15:01, B*51:01	vaccinated	yes	A*02/S269-277	yes	yes	yes
V9	m	61	A*02:01, B*08:01, B*15:01	vaccinated	yes	A*02/S269-277	yes	yes	yes
V10	m	32	A*03:01, A*30:01, B*13:02, B*35:01	vaccinated	no	-	yes	yes	yes
V11	f	53	A*01:01, A*24:02, B*08:01, B*27:05	vaccinated	yes	A*01/S865-873	yes	yes	yes
V12	f	38	A*02:01, A*68:01, B*15:01, B*40:01	vaccinated	no	-	yes	yes	yes
V13	m	36	A*01:01, A*26:01, B*07:02, B*27:05	vaccinated	no	-	yes	yes	yes
V14	m	34	A*02:01, A*03:01, B*07:02	vaccinated	no	-	yes	yes	yes
V15	m	30	A*23:01, A*26:01, B*44:03, B*45:01	vaccinated	no	-	no	yes	yes
V16	m	48	A*02:01, A*24:02, B*27:05, B*51:01	vaccinated	no	-	no	yes	yes
V17	m	31	A*0101, A*0201, B*1302, B*3502	vaccinated	no	-	yes	yes	yes
V18	f	30	A*01:01, A*33:01, B*14:02, B*57:01	vaccinated	no	-	yes	yes	yes
V19	m	43	A*03:01, A*32:01, B*07:02, B*40:02	vaccinated	no	-	yes	yes	yes
V20	f	36	A*02:01, A*68:01, B*15:01, B*55:01	vaccinated	no	-	yes	yes	yes
V21	m	27	A*02:01, A*29:02, B*45:01, B*51:01	vaccinated	no	-	yes	yes	yes
V22	f	28	A*02:01, A*31:01, B*07:02, B*45:01	vaccinated	no	-	yes	yes	yes
V23	m	32	A*01:01, A*03:01, B*08:01, B*18:01	vaccinated	no	-	yes	yes	yes
V24	f	28	A*02:01, B*07:02, B*08:01	vaccinated	no	-	yes	yes	yes
V25	f	27	A*02:01	vaccinated	yes	A*02/S269-277	no	yes	no
V26/BTI1	m	42	A*01:01, A*03:01, B*08:01, B*35:01	vaccinated / break	yes	A*01/S865-873	yes	yes	yes

V27/BTI2	m	49	A*01:01, A*11:01, B*08:01, B*15:01	vaccinated / breakthrough infection	yes	A*01/S865-873	yes	yes	yes
V28/BTI3	m	63	A*01:01, A*02:01, B*08:01, B*15:01	vaccinated / breakthrough infection	yes	A*01/S865-873<br>A*02/S269-277	yes	yes	yes
V29/BTI4	m	36	A*01:01, A*24:02, B*07:02, B*27:05	vaccinated / breakthrough infection	yes	A*01/S865-873	yes	yes	yes
V30/BTI5	m	33	A*02:01, A*24:02, B*27:05, B*27:07	vaccinated / breakthrough infection	yes	A*02/S269-277	yes	yes	yes
V31/BTI6	m	42	A*01:01, A*02:01, B*08:01, B*40:01	vaccinated / breakthrough infection	no	-	yes	yes	yes
BTI7	f	23	A*01:01, A*02:01, B*15:01, B*44:03	breakthrough infection	yes	A*01/S865-873<br>A*02/S269-277	yes	no	yes
BTI8	f	25	A*01:01, A*68:02, B*53:01, B*57:01	breakthrough infection	yes	A*02/S269-277	yes	no	yes
BTI9	m	23	A*02:01, B*27:05, B*35:01	breakthrough infection	yes	A*02/S269-277	yes	no	yes
BTI10	f	23	A*02:01, B*07:02, B*40:01	breakthrough infection	yes	A*02/S269-277	yes	no	yes
BTI11	m	26	A*02:01, A*23:01, B*40:01, B*44:03	breakthrough infection	yes	A*02/S269-277	yes	no	yes
BTI12	m	24	A*02:01, A*24:02, B*35:03	breakthrough infection	yes	A*02/S269-277	yes	no	yes
BTI13	f	43	A*02:01, B*27:05, B*44:02	breakthrough infection	yes	A*02/S269-277	no	no	no

Supplementary Table 2: List of antibodies

Antigen	Conjugate	Clone	Dilution	Isotype	Catalogue number	Manufacturer
CCR7	PE-CF594	150503	1:50	Mouse IgG2a	562381	BD Biosciences
CD4	BV786	L200	1:200	Mouse BALB/c IgG1, κ	563914	BD Biosciences
CD8	BUV395	RPA-T8	1:400	Mouse IgG1, κ	563795	BD Biosciences
CD8	BV510	SK1	1:100	Mouse BALB/c IgG1, κ	563919	BD Biosciences
CD8	BV421	RPA-T8	1:200	Mouse IgG1, k	562428	BD Biosciences
CD11a	BV510	HI111	1:25	Mouse IgG1, k	563479	BD Biosciences
CD28	BV421	CD28.2	1:100	Mouse C3H x BALB/c IgG1, κ	562613	BD Biosciences
CD38	APC-R700	HIT2	1:400	Mouse IgG1, κ	564980	BD Biosciences
CD39	BV650	TU66	1:33	Mouse IgG2b, κ	563681	BD Biosciences
CD45RA	BUV496	HI100	1:800	Mouse IgG2b, κ	750258	BD Biosciences
CD45RA	BUV737	HI100	1:800	Mouse IgG2b, κ	564442	BD Biosciences
CD95	APC	DX2	1:100	Mouse C3H	558814	BD Biosciences
CD107a	APC	H4A3	1:100	Mouse BALB/c IgG1, κ	560664	BD Biosciences
CD127	BUV737	HIL-7R-M21	1:50	Mouse IgG1, κ	612795	BD Biosciences
CD137	BV650	4B4-1	1:100	Mouse BALB/c IgG1, κ	564092	BD Biosciences
Granzyme B	PE-CF594	GB11	1:800	Mouse BALB/c IgG1, κ	562462	BD Biosciences
Granzyme B	BV510	GB11	1:50	Mouse BALB/c IgG1, κ	563388	BD Biosciences
IFN-γ	FITC	25723.11	1:8	Mouse IgG2b	340449	BD Biosciences
PD-1	BV605	EH12.1	1:50	Mouse IgG1, κ	563245	BD Biosciences
PD-1	PE-Cy7	EH12.1	1:200	Mouse IgG1, κ	561272	BD Biosciences
TNF	PE-Cy7	MAb11	1:400	Mouse IgG1, κ	557647	BD Biosciences
HLA-DR	BUV395	G46-6	1:200	Mouse IgG2a, κ	565972	BD Biosciences
BCL-2	BV421	100	1:200	Mouse IgG1	658709	BioLegend
CCR7	BV785	G043H7	1:50	Mouse IgG2a, κ	353230	BioLegend
CD57	BV605	QA17A04	1:100	Mouse IgG1, κ	393304	BioLegend
CXCR3	PerCP-Cy5.5	G025H7	1:33	Mouse IgG1, κ	353714	BioLegend
IL-2	PerCP-Cy5.5	MQ1-17H12	1:100	Rat IgG2a, κ	500322	BioLegend
Ki67	BV711	Ki-67	1:200	Mouse IgG1, κ	350516	BioLegend
Perforin	PerCP-Cy5.5	B-D48	1:200	Mouse IgG1	353314	BioLegend
Granzyme K	PE-Cy7	GM26E7	1:25	Mouse IgG1, κ	370516	BioLegend
Granulysin	APC	DH2	1:100	Mouse IgG1, κ	348010	BioLegend

TCF1	AlexaFluor488	C63D9	1:100	Rabbit IgG	6444	Cell Signaling
CD14	APC-eFluor780	61D3	1:400	Mouse IgG1, k	47-0149-42	eBioscience
CD19	APC-eFluor780	HIB19	1:400	Mouse IgG1, k	47-0199	eBioscience
CD27	FITC	O323	1:100	Mouse IgG1, k	11-0279	eBioscience
Eomes	PerCP-eFluor710	WD1928	1:10	Mouse IgG1, k	46-4877	eBioscience
KLRG1	BV711	13F12F2	1:50	Mouse IgG	67-9488-42	eBioscience
T-bet	PE-Cy7	4B10	1:200	Mouse IgG1, k	25-5825	eBioscience
TOX	eFluor660	TRX10	1:100	Rat IgG2a, k	50-6502	eBioscience
viability dye	eFluor780	65-0865	1:400		65-0865	eBioscience
