# Supplementary material for: COVID-19 mRNA booster vaccine induces transient CD8+ T effector cell responses while conserving the memory pool for subsequent reactivation
Source: Nat Commun. 2022 Aug 8;13:4631. doi: 10.1038/s41467-022-32324-x (PMC9358914; doi:10.1038/s41467-022-32324-x)
Supplement: Supplementary file 3 — Reporting Summary [file 41467_2022_32324_MOESM3_ESM.pdf]

nature research

Corresponding author(s): Maike Hofmann
Christoph Neumann-Haefelin
Robert Thimme

Last updated by author(s): Jun 27, 2022

Reporting Summary

Nature Research wishes to improve the reproducibility of the work that we publish. This form provides structure for consistency and transparency in reporting. For further information on Nature Research policies, see our Editorial Policies and the Editorial Policy Checklist.

Statistics

For all statistical analyses, confirm that the following items are present in the figure legend, table legend, main text, or Methods section.

n/a	Confirmed	
☐	☒	The exact sample size (*n*) for each experimental group/condition, given as a discrete number and unit of measurement
☐	☒	A statement on whether measurements were taken from distinct samples or whether the same sample was measured repeatedly
☐	☒	The statistical test(s) used AND whether they are one- or two-sided<br>*Only common tests should be described solely by name; describe more complex techniques in the Methods section.*
☐	☒	A description of all covariates tested
☐	☒	A description of any assumptions or corrections, such as tests of normality and adjustment for multiple comparisons
☐	☒	A full description of the statistical parameters including central tendency (e.g. means) or other basic estimates (e.g. regression coefficient) AND variation (e.g. standard deviation) or associated estimates of uncertainty (e.g. confidence intervals)
☐	☒	For null hypothesis testing, the test statistic (e.g. *F*, *t*, *r*) with confidence intervals, effect sizes, degrees of freedom and *P* value noted<br>*Give P values as exact values whenever suitable.*
☐	☒	For Bayesian analysis, information on the choice of priors and Markov chain Monte Carlo settings
☐	☒	For hierarchical and complex designs, identification of the appropriate level for tests and full reporting of outcomes
☒	☐	Estimates of effect sizes (e.g. Cohen's *d*, Pearson's *r*), indicating how they were calculated

Our web collection on statistics for biologists contains articles on many of the points above.

Software and code

Policy information about availability of computer code

Data collection	All software used to perform data collection are described in the methods section of the manuscript or the supportive information. Multiparametric Flow cytometry data was collected by FACSDiva software and CytExpert Software. ELISA data was collected by Magellan Software (version 2.2).
Data analysis	All codes used to perform bioinformatic analyses are described in the methods section of the manuscript or the supportive information. Multiparametric Flow cytometry data was analyzed using FlowJo software version 10.6.2 (Treestar, Becton Dickinson). The visualization of multiparametric flow cytometry data was done with R version 4.1.1 using the Bioconductor (version: Release (3.11)) CATALYST package (Crowell H, Zanotelli V, Chevrier S, Robinson M (2020). CATALYST: Cytometry dATa anALYSis Tools. R package version 1.12.2, https://github.com/HelenaLC/CATALYST). The R code data is available at https://github.com/sagar161286/SARSCoV2_specific_CD8_Tcells. Visualization and statistical analysis was performed using GraphPad 9.3.1 software. Sequence homology analyses were performed in Geneious Prime 2020.0.3 (https://www.geneious.com/) using Clustal Omega 1.2.2 alignment with default settings.

For manuscripts utilizing custom algorithms or software that are central to the research but not yet described in published literature, software must be made available to editors and reviewers. We strongly encourage code deposition in a community repository (e.g. GitHub). See the Nature Research guidelines for submitting code & software for further information.

Data

Policy information about availability of data

All manuscripts must include a data availability statement. This statement should provide the following information, where applicable:

- Accession codes, unique identifiers, or web links for publicly available datasets
- A list of figures that have associated raw data
- A description of any restrictions on data availability

The raw values for charts and graphs are available in the Source Data file whenever possible. All requests for additional raw (especially flow cytometry data) and materials are promptly reviewed by the University of Freiburg Center for Technology Transfer to verify if the request is subject to any intellectual property or confidentiality obligations. Donor-related data not included in the paper were generated as part of clinical examination and may be subject to donor confidentiality. Any data and materials that can be shared will be released via a Material Transfer Agreement.

Field-specific reporting

Please select the one below that is the best fit for your research. If you are not sure, read the appropriate sections before making your selection.

- [x] Life sciences
- [ ] Behavioural & social sciences
- [ ] Ecological, evolutionary & environmental sciences

For a reference copy of the document with all sections, see nature.com/documents/nr-reporting-summary-flat.pdf

Life sciences study design

All studies must disclose on these points even when the disclosure is negative.

Sample size	Patients were recruited and patient material was banked at the University Hospital Freiburg. Inclusion criteria were: (1) 38 subjects that received up to four mRNA vaccinations with the mRNA vaccine Comirnaty, Biontech/Pfizer or Spikevax/Moderna and (2) 13 subbjects with mild SARS-CoV-2 breakthrough infection following three doses of a mRNA vaccination, SARS-CoV-2 infection was confirmed by positive PCR testing from oropharyngeal swab and/or SARS-CoV-2 spike IgG positive antibody testing.<br>These numbers are comparable to many other studies in the research field of human immunology and proved to be sufficient for the generation of reproducible results.
Data exclusions	For flow cytometrical analysis, cell populations containing less than 5 cells were excluded. This data exclusion strategy has been applied and validated previously by our group to gain reproducible results in studies investigating virus-specific CD8+ T cells in human viral infections.
Replication	Analyses were performed in independent experiments. Findings were reproducible. Flow cytometry analysis: 7 longitudinally analyzed vaccinees for A*01/S865 (n=6 at Baseline (BL), n=5 after 6-10 days post 1st vaccination, n=5 11-21 days post 1st vaccination, n=6 after 3-10 days post 2nd vaccination, n=5 after 11-20 days post 2nd vaccination, n=6 after 28-35 days post 2nd vaccination, n=3 after 50-60 days post 2nd vaccination, n=3 after 80-90 days post 2nd vaccination, n=7 after 210-310 days post 2nd vaccination, n=5 after 3-10 days post 3rd vaccination, n=6 after 11-20 days post 3rd vaccination, n=6 after 21-30 days post 3rd vaccination, n=6 after 31-42 days post 3rd vaccination, n=4 after 43-55 days post 3rd vaccination, n=3 after 69-82 days post 3rd vaccination, n=5 after 91-120 days post 3rd vaccination, n=2 after 5-10 days post 4th vaccination, n=2 after 11-20 days post 4th vaccination, n= 1 after 20-25, 26-30, 90-100 days post 4th vaccination) and 8 longitudinally analyzed vaccinees for A*02/S269 (n=7 at BL, n=3 after 6-10 days post 1st vaccination, n=7 11-21 days post 1st vaccination, n=7 after 3-10 days post 2nd vaccination, n=6 after 11-20 days post 2nd vaccination, n=7 after 28-36 days post 2nd vaccination, n=3 after 50-60 days post 2nd vaccination, n=3 after 80-90 days post 2nd vaccination, n=2 after 109-131 days post 2nd vaccination, n=5 after 230-270 days post 2nd vaccination, n=4 after 3-10 days post 3rd vaccination, n=5 after 11-20 days post 3rd vaccination, n=6 after 21-30 days post 3rd vaccination, n=5 after 31-49 days post 3rd vaccination, n=4 after 50-75 days post 3rd vaccination, n=6 after 82-105 days post 3rd vaccination, n=1 after 5-10 days post 4th vaccination, n=2 after 11-20 days post 4th vaccination, n=2 after 21-30 days post 4th vaccination, n=1 after 30-35, 45-50, 60-65 days post 4th vaccination) in independent experiments (Figures 1a, 1a, 2c, 3a, 3b, 3c, 3d, 4a, 4b, 4c, 4d, 4e, 4f, 5a, 5b, 6c and Suppl. Fig. 2a, 4b, 5a, 5b, 5c, 6a, 6b, 7c, 7d, 7e, 8a, 9a, 9b); 6 longitudinally analyzed subjects following a mild course of SARS-CoV-2 breakthrough infection after three doses of a mRNA vaccine (Comirnaty/Biontech or Spikevax/Moderna) for A*01/S865 (n=5 for Omicron with n=5 after 3-7 days post symptom onset (dps), n=4 after 8-14 dps, n=3 after 15-21 dps, n=3 after 22-30 dps, n=3 after 31-45 dps, and n=1 for Delta infection with n=1 after 1-7, 8-14, 35-40, 60-65, 80-85 dps) and 8 longitudinally analyzed subjects following a mild course of SARS-CoV-2 breakthrough infection after three doses of a mRNA vaccine (Comirnaty/Biontech or Spikevax/Moderna) for A*02/S269 (n= 7 for Omicron with n=6 after 3-7 days post symptom onset (dps), n=3 after 8-14 dps, n=5 after 15-21 dps, n=3 after 22-30 dps, n=3 after 31-45 dps, and n=1 for Delta infection with n=1 after 1-7, 8-14, 35-40, 60-65, 80-85 dps) in independent experiments (Figure 5a, 5b, 6a, 6c and Suppl. Fig. 8a, 9b); n=6 analyzed pre-pandemic samples (Suppl. Fig. 1c) in independent experiments.<br>ELISA and NT analysis: 28 and 26 longitudinally analyzed vaccinees that received three or four doses of a mRNA vaccination (Comirnaty/ Biontech or Spikevax/Moderna) were tested for B.1, B.1.617 and B.1.1.529 neutralizing titers (n=25 before 3rd vaccination and n=11 after 1-5, n=13 after 6-10, n=18 after 11-15, n=8 after 16-20, n=13 after 21-25, n=17 after 26-30, n=15 after 31-40, n=5 after 41-45, n=9 after 51-60, n=5 after 61-70, n=4 after 71-80, n=4 after 81-90, n=6 after 91-100, n=4 after 101-110, n=2 after 111-120, n=2 after 131-140, n=1 after 161-170 days post 3rd vaccination, and n=2 after 1-10 and 11-20 days, n=1 after 21-25 days, n=2 after 26-35 days, n=1 after 45-50 and 61-65 days post 4th vaccination) and for anti-SARS-CoV-2-S1 IgG (n=22 before 3rd vaccination and n=12 after 1-5, n=11 after 6-10, n=16 after 11-15, n=8 after 16-20, n=12 after 21-25, n=17 after 26-30, n=14 after 31-40, n=4 after 41-45, n=5 after 45-50, n=9 after 51-60, n=5 after 61-70, n=3 after 71-80, n=4 after 81-90, n=6 after 91-100, n=3 after 101-110, n=2 after 111-120, n=3 after 131-140, n=1 after 161-170 days post 3rd vaccination, n=2 after 1-10, 11-20, 21-25, 26-30, 31-40, 41-50, 51-60 days post 4th vaccination), respectively, and 12 longitudinally analyzed vaccinees with mild SARS-CoV-2 breakthrough infection after a third dose of a mRNA vaccination (Comirnaty/Biontech or Spikevax/Moderna) for B.1, B.1.617 and B.1.1.529 neutralizing titers (n=11 for Omicron infection with n=9 after 1-7 dps, n=7 after 8-14 dps, n=6 after 15-21 dps, n=4 after 22-28 dps, n=4 after 29-35 dps, n=2 after 40-50 dps, and n=1 for Delta infection with n=1 after 1-7, 8-14, 15-20, 35-40, 60-65, 80-85

dps) in independent experiments (Figure 2a, 2b, 6d, Suppl. Fig. 3c).

Randomization

Vaccinated donors and donors with a history of SARS-CoV-2 breakthrough infection were selected based on availability and HLA-typing. The covariates age and gender are well-documented: Median age of vaccinated donors was 36 years, donors with a history of SARS-CoV-2 breakthrough infection was 33 years. The gender ratio of vaccinated donors was m/f: 18/13, donors with a history of SARS-CoV-2 breakthrough infection was m/f: 9/4.

Blinding

Blinding was not applied. Non-objective parameters were not included in the study design. Due to standardized analyses of the flow cytometric data set, biased analysis can be excluded.

Reporting for specific materials, systems and methods

We require information from authors about some types of materials, experimental systems and methods used in many studies. Here, indicate whether each material, system or method listed is relevant to your study. If you are not sure if a list item applies to your research, read the appropriate section before selecting a response.

Materials & experimental systems

n/a	Involved in the study
☐	☒ Antibodies
☒	☐ Eukaryotic cell lines
☒	☐ Palaeontology and archaeology
☒	☐ Animals and other organisms
☐	☒ Human research participants
☒	☐ Clinical data
☒	☐ Dual use research of concern

Methods

n/a	Involved in the study
☒	☐ ChIP-seq
☐	☒ Flow cytometry
☒	☐ MRI-based neuroimaging

Antibodies

Antibodies used

BD Biosciences:
anti-CCR7-PE-CF594 (150503, 1:50), mouse, Cat# 562381
anti-CD4-BV786 (L200, 1:200), mouse, Cat# 563914
anti-CD8-BUV395 (RPA-T8, 1:400), mouse, Cat# 563795
anti-CD8-BV510 (SK1, 1:100), mouse, Cat# 563919
anti-CD8-BV421 (RPA-T8, 1:200), mouse, Cat# 562428
anti-CD11a-BV510 (HI111, 1:25), mouse, Cat# 563479
anti-CD28-BV421 (CD28.2, 1:100), mouse, Cat# 562613
anti-CD38-APC-R700 (HIT2, 1:400), mouse, Cat# 564980
anti-CD39-BV650 (TU66, 33:1), mouse, Cat# 563681
anti-CD45RA-BUV496 (HI100, 1:800), mouse, Cat# 750258
anti-CD45RA-BUV737 (HI100, 1:800), mouse, Cat# 564442
anti-CD95-APC (DX2, 1:100), mouse, Cat# 558814
anti-CD107a-APC (H4A3, 1:100), mouse, Cat# 560664
anti-CD127-BUV737 (HIL-7R-M21, 1:50), mouse, Cat# 612795
anti-CD137-BV650 (4B4-1, 1:100), mouse, Cat# 564092
anti-Granzyme B-PE-CF594 (GB11, 1:800), mouse, Cat# 562462
anti-Granzyme B-BV510 (GB11, 1:50), mouse, Cat# 563388
anti-IFN-γ-FITC (25723.11, 1:8), mouse, Cat# 340449
anti-PD-1-BV605 (EH12.1, 1:50), mouse, Cat# 563245
anti-PD-1-PE-Cy7 (EH12.1, 1:200), mouse, Cat# 561272
anti-TNF-PE-Cy7 (Mab11, 1:400), mouse, Cat# 557647
anti-HLA-DR-BUV395 (G46-6, 1:200), mouse, Cat# 565972

BioLegend:
anti-BCL-2-BV421 (100, 1:200), mouse, Cat# 658709
anti-CCR7-BV785 (G043H7, 1:50), mouse, Cat# 353230
anti-CD57-BV605 (QA17A04, 1:100), mouse, Cat# 393304
anti-CXCR3-PerCP-Cy5.5 (G025H7, 1:33), mouse, Cat# 353714
anti-IL-2-PerCP-Cy5.5 (MQ1-17H12, 1:100), rat, Cat# 500322
anti-Ki67-BV711 (Ki-67, 1:200), mouse, Cat# 350516
anti-Perforin-PerCP-Cy5.5 (B-D48, 1:200), mouse, Cat# 353314
anti-Granzyme K-PE-Cy7 (GM26E7, 1:25), mouse, Cat# 370516
anti-Granulysin-APC (DH2, 1:100), mouse, Cat# 348010

Cell Signaling:
anti-TCF1-AlexaFluor488 (C63D9, 1:100), rabbit, Cat# 6444

eBioscience:
anti-CD14-APC-eFluor780 (61D3, 1:400), mouse, Cat# 47-0149-42
anti-CD19-APC-eFluor780 (HIB19, 1:400), mouse, Cat# 47-0199
anti-CD27-FITC (0323, 1:100), mouse, Cat# 11-0279
anti-EOMES-PerCP-eF710 (WD1928, 1:10), mouse, Cat# 46-4877
anti-KLRG1-BV711 (13F12F2, 1:50), mouse, Cat# 67-9488-42
anti-T-BET-PE-Cy7 (4B10, 1:200), mouse, Cat# 25-5825
anti-TOX-eFluor660 (TRX10, 1:100), rat, Cat# 50-6502
Viability Dye (APC-eFluor780, 1:400) Cat# 65-0865

Validation

Standardized analysis in different cohorts, antibody titration on PBMCs including unstained controls, comparisons of different antibody clones and conjugates, and validated by publications:
BCL-2, clone 100, flow cytometry: antibody titration on PBMCs; validated with respect to differential expression of naïve and non-naïve T cell subpopulations (PMID: 34320609)
CCR7, clone G043H7 and 150503, flow cytometry: antibody titration on PBMCs; control clone G043H7; validated with respect to differential expression of naïve and non-naïve T cell subpopulations (PMID: 8383238, 33296701)
CD4, clone L200, flow cytometry: antibody titration on PBMCs; control clones SK3; using B cells as negative control (PMID: 1713585)
CD8, clone SK1 and RPA-T8, flow cytometry: antibody titration on PBMCs; control clone GHI/75; using B cells as negative control (PMID: 35654046, 35572194, 35474871)
CD11a, clone HI111, flow cytometry: antibody titration on PBMCs, control clones TS2/4 and G43-25B; validated with respect to differential expression of activated and non-activated T cell subpopulations (PMID: 1709678)
CD14, clone 61D3, flow cytometry: antibody titration on PBMCs; control clones M5E2 and MϕP9; using T cell populations as negative control (PMID: 34321489)
CD19, clone HIB19, flow cytometry: antibody titration on PBMCs; control clone SJ25C1; using T cell populations as negative control (PMID: 34133950)
CD27, clone O323, flow cytometry: antibody titration on PBMCs; control clone L128; validated with respect to differential expression of naïve and non-naïve T cell subpopulations (PMID: 31839985)
CD28, clone CD28.2, flow cytometry: antibody titration on PBMCs; control clone B-T3; validated with respect to differential expression of naïve and non-naïve T cell subpopulations (PMID: 7522010)
CD38, clone HIT2, flow cytometry: antibody titration on PBMCs; control clone HIT2.1; validated with respect to differential expression of naïve and non-naïve T cell subpopulations (PMID: 9551996)
CD39, clone TU66, flow cytometry: antibody titration on PBMCs; control clones eBioA1 and A1; validated with respect to differential expression of naïve and non-naïve T cell subpopulations (PMID: 17449799)
CD45RA, clone HI100, flow cytometry: antibody titration on PBMCs; validated with respect to differential expression of naïve and non-naïve T cell subpopulations (PMID: 33948610, 34726156)
CD57, clone QA17A04, flow cytometry: antibody titration on PBMCs; control clone NK-1; validated with respect to differential expression of naïve and non-naïve T cell subpopulations (PMID: 32967958)
CD95, clone DX2, flow cytometry: Titration on fresh PBMCs and in vitro activated B cells; validated with respect to differential expression of naïve and non-naïve T cell subpopulations (PMID: 7523573)
CD107a, clone H4A3, flow cytometry: antibody titration on PBMCs; validated with respect to differential expression of activated and non-activated T cell subpopulations (PMID: 12186851)
CD127, clone HIL-7R-M21, flow cytometry: antibody titration on PBMCs; control clone eBioRDR5; validated with respect to differential expression of naïve and non-naïve T cell subpopulations (PMID: 10390077)
CD137, clone 4B4-1, flow cytometry: antibody titration on PBMCs; validated with respect to differential expression of activated and non-activated T cell subpopulations (PMID: 8612300)
CXCR3, clone G025H7, flow cytometry: antibody titration on PBMCs; control clone 1C6/CXCR3; validated with respect to differential expression of activated and non-activated T cell subpopulations (PMID: 30127434)
EOMES, clone WD1928, flow cytometry: antibody titration on PBMCs; validated with respect to differential expression of naïve and non-naïve T cell subpopulations (PMID: 33668625)
Granulysin, clone DH2, flow cytometry: antibody titration on PBMCs; validated with respect to differential expression of activated and non-activated T cell subpopulations (PMID: 10727456)
Granzyme B, clone GB11, flow cytometry: antibody titration on PBMCs; polyclonal antibody as control; validated with respect to differential expression of activated and non-activated T cell subpopulations (PMID: 9348298)
Granzyme K, clone GM26E7, flow cytometry: antibody titration on PBMCs; validated with respect to differential expression of activated and non-activated T cell subpopulations (no reference available)
HLA-DR, clone G46-6, flow cytometry: antibody titration on PBMCs: validated with respect to differential expression on activated and non-activated T cell subpopulations (PMID: 11390437)
IFNɣ, clone 25723.11, flow cytometry: antibody titration on PBMCs; control clone 4S.B3; validated with respect to differential expression of activated and non-activated T cell subpopulations (PMID: 35484232)
IL-2, clone MQ1-17H12, flow cytometry: antibody titration on PBMCs; validated with respect to differential expression of activated and non-activated T cell subpopulations (PMID: 28759590)
KLRG1, clone 13F12F2, flow cytometry: antibody titration on PBMCs; validated with respect to differential expression of naïve and non-naïve T cell subpopulations (PMID: 34320609)
Ki67, clone Ki67, flow cytometry: antibody titration on PBMCs; control clone B56; validated with respect to differential expression of naïve and non-naïve T cell subpopulations (PMID: 30661959)
PD-1, clone EH12.1, flow cytometry: antibody titration on PBMCs; control clone eBioJ105; validated with respect to differential expression of naïve and non-naïve T cell subpopulations (PMID: 12517932)
Perforin, clone B-D48, flow cytometry: antibody titration on PBMCs; validated with respect to differential expression of activated and non-activated T cell subpopulations (PMID: 30503208)

TNF, clone MAb11, flow cytometry: antibody titration on PBMCs; validated with respect to differential expression of activated and non-activated T cell subpopulations (PMID: 9328582)
TCF-1, clone C63D9, flow cytometry: antibody titration on PBMCs; control clone 7F11A10; validated with respect to differential expression of naïve and non-naïve T cell subpopulations (PMID: 34795301)
T-BET, clone 4B10, flow cytometry: antibody titration on PBMCs; control clones 4B10; validated with respect to differential expression of naïve and non-naïve T cell subpopulations (PMID: 30970256)
TOX, clone TRX10, flow cytometry: antibody titration on PBMCs; control clone REA473; validated with respect to differential expression of naïve and non-naïve T cell subpopulations (PMID: 28239338)
Viability Dye, clone 65-0865, were titrated on PBMCs; validated with respect to differential staining of live and dead cell populations (PMID: 34320609)

Human research participants

Policy information about studies involving human research participants

Population characteristics	38 subjects (median age 36 years) that received up to four vaccinations with the mRNA vaccine Comirnaty/Biontech or Spikevax/Moderna and 13 subjects (median 33 years) with mild SARS-CoV-2 breakthrough infection carrying the HLA alleles A*01:01 and/or -A*02:01 were recruited at the University Hospital Freiburg. The gender ratio of vaccinated donors was m/f: 18/13 and donors with mild SARS-CoV-2 breakthrough infection after three doses of mRNA vaccine was m/f: 9/4. A detailed overview of all patient characteristics is depicted in Suppl. Table 1.
Recruitment	Study participants voluntarily donated blood at he University Hospital Freiburg after written informed conset was obtained. Blood samples were banked and retrospectively selected according to the following inclusion criteria: All donors were vaccinated with a mRNA vaccine and were positive for either HLA-A*01:01 or HLA-A*02:01 and lacked comorbidities. Participants did not receive compensation.
Ethics oversight	Written informed consent was obtained from all participants and the study was conducted according to federal guidelines, local ethics committee regulations (Albert-Ludwigs-Universität, Freiburg, Germany; vote #: 322/20, #21-1135 and 315/20) and the Declaration of Helsinki (1975).

Note that full information on the approval of the study protocol must also be provided in the manuscript.

Flow Cytometry

Plots

Confirm that:

- [x] The axis labels state the marker and fluorochrome used (e.g. CD4-FITC).
- [x] The axis scales are clearly visible. Include numbers along axes only for bottom left plot of group (a 'group' is an analysis of identical markers).
- [x] All plots are contour plots with outliers or pseudocolor plots.
- [x] A numerical value for number of cells or percentage (with statistics) is provided.

Methodology

Sample preparation	Cryopreserved isolated human PBMCs were thawed and prepared for flow cytometry or in vitro expansion described in the methods section
Instrument	FACSCanto II, LSRFortessa (BD, Germany), Cytek Aurora (Cytek) or CytoFLEX (Beckman Coulter), Tecan (LifeScience)
Software	FlowJo_v10.6.2 (Treestar), R version 4.0.2 using the Bioconductor (version: Release (3.11)) .
Cell population abundance	Abundance of SARS-CoV-2-specific T cells are low (<10^-4 %)
Gating strategy	CD8+ T cells: Lymphocytes gated on FSC-A and SSC-A, doublet exclusion on FSC-W and FSC-H and SSC-W and SSC-H, exclusion of dead cells, B cells and monocytes, gating on CD8+ cells, gating of SARS-CoV-2-specific CD8+ T cells via tetramers described in methods part. Naive cells (CD45RA+, CCR7+) were excluded on demand.

- [x] Tick this box to confirm that a figure exemplifying the gating strategy is provided in the Supplementary Information.
